# A Novel Classification and Scoring Method Based on Immune-Related Transcription Factor Regulation Patterns in Gastric Cancer

**DOI:** 10.3389/fonc.2022.887244

**Published:** 2022-05-17

**Authors:** Gang-Jian Wang, Long-Tao Huangfu, Xiang-Yu Gao, Xue-Jun Gan, Xiao-Fang Xing, Jia-Fu Ji

**Affiliations:** ^1^ Key Laboratory of Carcinogenesis and Translational Research (Ministry of Education), Division of Gastrointestinal Cancer Translational Research Laboratory, Peking University Cancer Hospital, Beijing, China; ^2^ Department of Gastrointestinal Surgery, Peking University Cancer Hospital, Beijing, China

**Keywords:** gastric cancer, transcription factor, immune microenvironment, immunogenicity, immunotherapy transcription factor, immunotherapy

## Abstract

**Background:**

Transcription factors (TFs) play a crucial role in tumorigenesis and anti-tumor immunity. However, the potential role of large-scale transcription factor regulation patterns in the progression in gastric cancer (GC) is unknown.

**Methods:**

We comprehensively assessed the relevance of immune-related TF (IRTF) regulation patterns in anti-tumor immunity and immunotherapy in 1,136 gastric cancer (GC) patients, and evaluated the IRTF score based on IRTF regulation patterns using random forests.

**Results:**

Two distinct IRTF regulation patterns were identified, which demonstrating the distinct characteristics in clinical phenotypes, tumor immune microenvironment (TIME), immunogenicity and prognosis in GC. Subsequently, the IRTF score was established to quantify the IRTF regulation pattern for each GC patient. Analysis of large conventional therapy cohorts showed low IRTF score was associated with a better prognosis. In addition, analysis of multiple immunotherapy cohorts showed low IRTF score was also linked to enhanced response to immunotherapy.

**Conclusion:**

TF regulation patterns were found to play an important role in the complex immune regulatory relationships in GC. Evaluation of the IRTF regulation patterns in patients will enhance our understanding of immune specificities, and thus, provide effective strategies for personalized therapy.

**Graphical Abstract:**
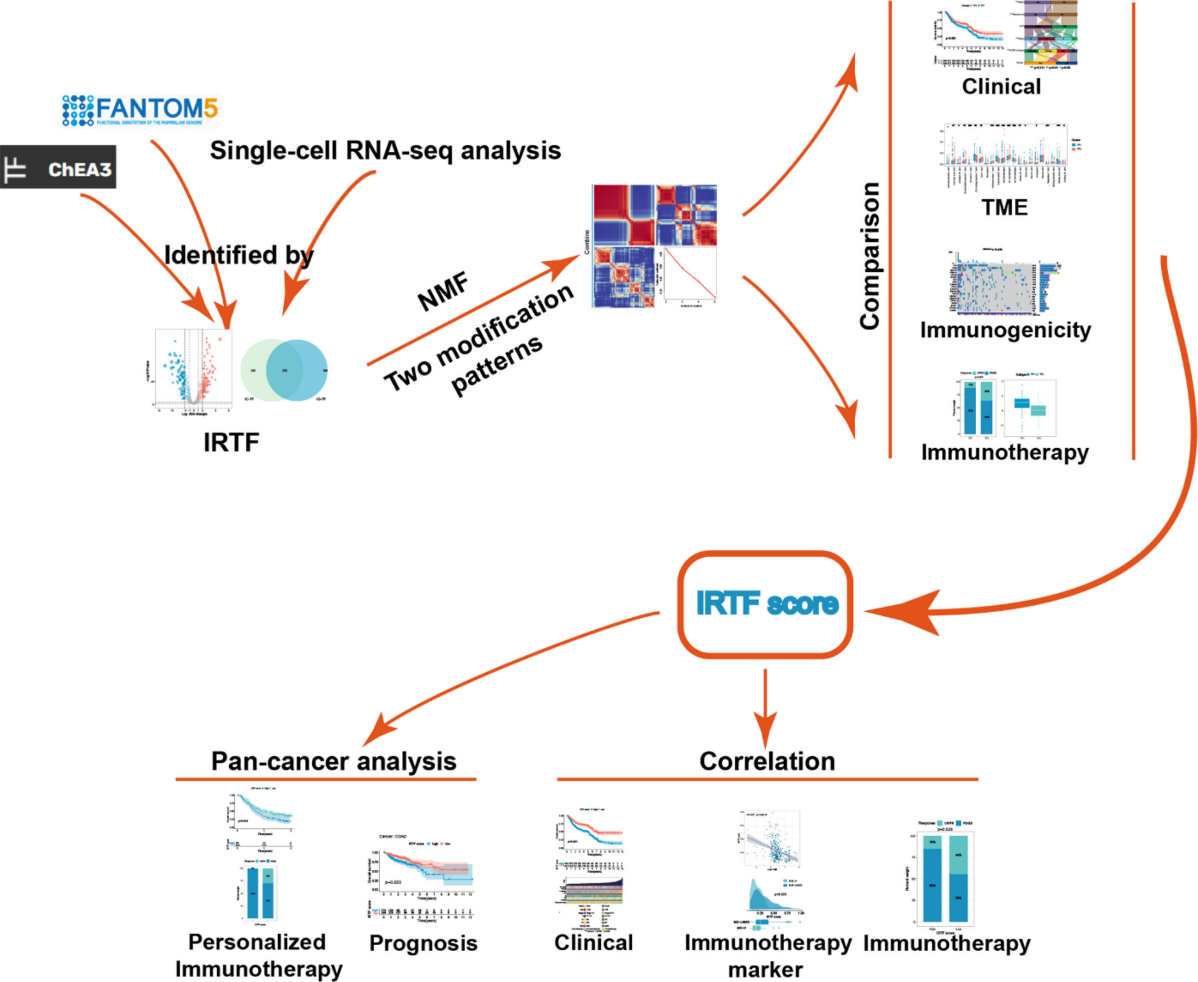


## Introduction

The latest data from the Global Cancer Statistics (2020) showed that gastric cancer (GC) is the fifth most common malignancy in the world, with the highest incidence in East Asia (1). Despite advances in diagnostic techniques, improved surgical procedures, and effective chemotherapeutic and immunotherapeutic agents that have significantly improved survival in GC over the past decade, GC, unfortunately, remains the fourth leading cause of cancer-related deaths (2, 3). The prognosis of GC not only depends on the stage of the disease but also involve specific molecular, biological, and immunological characteristics (4–6).

Immunotherapy has become an important therapeutic strategy that can inhibit tumor growth by activating the body’s anti-tumor immune response and curbing the immune escape of tumor cells (7). But unfortunately, only a small percentage of patients with solid tumors can benefit from it (8). there is an urgent need for a comprehensive understanding of tumor-immune interactions. Transcription factors (TFs), as a special group of biomolecules, play an important role in tumorigenesis and progression (9, 10). In recent years, with a better understanding of tumor heterogeneity, diversity and complexity, a large number of studies have revealed the involvement of TFs in the regulation of tumor immune microenvironment, immune escape and anti-tumor immunity (11–14). Mayes et al. showed that BPTF depletion upregulates the antigen processing genes Psmb8, Psmb9, Tap1, and Tap2, thereby enhancing tumor immunogenicity and anti-tumor immunity (14). Ni et al. showed that HIF-1α can regulate the anti-tumor activity of NK cells using single-cell sequencing (15). Gautam et al. found that overexpression of c-Myb not only preserves the stemness of CD8+ T cells but also promotes their proliferation, thereby enhancing anti-tumor immunity and establishing long-term immune memory (16). Kohanbash et al. showed that STAT1 affects cytotoxic T lymphocyte recruitment by regulating the expression of the chemokine CXCL10 (17).

Due to limitations of classical experimental science, these studies were limited to one or two TFs and cell types, but the interaction between tumorigenesis and anti-tumor immunity is a synergistic process in which multiple TFs are activated. Therefore, We first defined IRTFs through the transcription factor enrichment analysis of the ChEA3 database and the differential analysis of FAMTOM5. Subsequently, we identified the IRTF regulation pattern by NMF and comprehensively recognized the prognostic and immunological landscape mediated by the regulation patterns of IRTFs. Finally, a scoring system was developed to quantify this pattern and its prognostic value were further analyzed.

## Methods

### Dataset Sources and Preprocessing

Dataset sources in present study were summarized in **Supplementary Methods**. **Table 1** summarizes information on cell lines from the FANTOM5 database and **Table 2** summarizes the public transcriptome expression data included in this study.

**Table 1:** Immune cell/Tumor cell samples in FANTOM5.

Immune cell or tumor cell type	Number of samples	In total
Basophil	3	110
Monocyte	42	
B cell	11	
T cell, CD4+	3	
T cell	24	
T cell, CD8+	8	
Dendritic cell, myeloid, immature	2	
Eosinophil	3	
Macrophage	3	
Natural killer cell	3	
Neutrophil	6	
T cell, gamma-delta	2	
Adrenal gland	3	194
**ANATOMICAL SYSTEM**	7	
Bile duct	2	
Bladder	2	
Bone	4	
Bone marrow	3	
Brain	10	
Breast	3	
Cervix	11	
Chorioamniotic membrane	1	
Colon	2	
Duodenum	1	
Endometrium	4	
Esophagus	1	
Eye	2	
Gall bladder	2	
Gum	2	
Hair follicle	1	
Intestine	1	
Kidney	9	
Liver	8	
Lung	23	
Mediastinum	1	
Mesothelium	17	
Neck	1	
Ovary	8	
Palate	1	
Pancreas	9	
Pelvis	2	
Peripheral nervous system	1	
Pharynx	1	
Placenta	1	
Prostate	2	
Rectum	1	
Retroperitoneum	2	
Sinus	1	
Skeletal muscle	2	
Skin	7	
Small intestine	1	
Stomach	10	
Synovium	1	
Testis	5	
Thorax	1	
Thymus	1	
Thyroid	5	
Tongue	2	
Unclassifiable	2	
Uterus	5	
Vulva	1	
Nasal septum	1	

**Table 2:** Basic information of datasets included in this study.

Cancer type	Accession number/Source	Number of patients	Survival data	Response data
STAD	GEO: GSE15459	200	OS	–
	GEO: GSE34942	56	OS	–
	GEO: GSE57303	70	OS	–
	GEO: GSE62254(ACRG)	300	OS/RFS	–
	PUCH	198 (176 with dMMR)	OS	–
	TCGA-STAD	375	OS	–
	ERP107734	45	–	–

READ	GSE87211	203	OS	–
COAD	GSE38832	122	OS	–
	GSE17538	238	OS	–
	GSE39582	585	OS	–
PAAD	GSE28735	45	OS	–
	GSE57495	63	OS	–
	GSE62452	65	OS	–
	GSE71729	145	OS	–
LIHC	LIRI	232	OS	–

ACC, UVM, THYM, LUNG, SARC, AECA, KDNY, CHOL, UCEC, PANC, OV, BRCA, STAD, COLO, GCT, SKCM, HNSC, ESCA, CERV, LYMP	Pender cohort	98	OS	CR, PR, SD, PD, NCB, DCB
UC	Mvigor210	298	OS	CR, PR, SD, PD
SKCM	GSE91061	105	–	CR, PR, SD, PD
SKCM	GSE78220	27	OS	CR, PR, SD, PD
SKCM	PRJEB23709	73	OS	CR, PR, SD, PD
SKCM	TCGA: SKCM (Immunotherapy)	70	OS	CR, PR, SD, PD
CLL	GSE148476	50	–	Good outcome, Poor outcome
BRCA	GSE173839	71	–	CR, NCR
Mesothelioma	GSE63557	20	–	Response, No response

### ChEA3 Regulation Factor (TF) Enrichment Analysis

ChEA3 (https://maayanlab.cloud/chea3/index.html#top) is a database for TF enrichment analysis of 1,632 human TFs. It integrates ENCODE (https://www.encodeproject.org/), ReMap (http://pedagogix-tagc.univ-mrs.fr/remap/), and several independently published CHIPseq data. Additionally, it integrates the regulation factor co-expression data with RNAseq data from GTEx (https://gtexportal.org/home/), TCGA, and ARCHS4 (https://maayanlab.cloud/archs4/). TF co-expression analysis of the genes in the Enrichr (https://maayanlab.cloud/Enrichr/) can also be integrated. A smaller mean rank of TF indicates higher confidence in the prediction (18). The list of immune-related genes was obtained from the Immport database (https://www.immport.org/).

### Identification of the Regulation Patterns of Immune-Related TFs (IRTFs)

Five microarray cohorts (GSE15459, GSE34942, GSE57303, GSE62254, and PUCH) were merged to form a combined cohort, and the “sva” package was used to correct for batch effects on different datasets. Based on the IRTFs, we performed non-negative matrix factorization (NMF) regulation patterning using the NMF R package to identify the different IRTF regulation patterns. We varied the number of regulation patterns (k) from 2 to 5 and selected the value of k (as the best number of regulation patterns) that resulted in the maximum cophenetic correlation coefficient. The above steps were repeated independently for the TCGA-STAD cohort. SubMap analysis (Gene Pattern modules) was used to evaluate the similarity of regulation patterns between independent cohorts based on the full mRNA expression profiles.

### Estimation of Immune Cell Abundancein TIME

CIBERSORT, a powerful algorithm, was used to estimate the abundance of 22 immune cells from bulk tumor samples relying on a signature containing 547 genes (19), including naive B cells, memory B cells, plasma cells, resting/activated dendritic cells, resting/activated NK cells, resting/activated mast cells, eosinophils, neutrophils, monocytes, M0, M1, and M2 macrophages, CD8+ T cells, regulatory T cells (Tregs), resting/activated memory CD4+ T cells, follicular helper T cells, naive CD4+ T cells, and gamma delta T cells. To ensure the accuracy of estimation, we only retained samples with p-values < 0.01.

### Gene Set Variation Analysis (GSVA)

To assess the differences in the biological processes between different TF regulation patterns, we performed the GSVA enrichment analysis based on the “GSVA” R package. GSVA algorithm is a method of gene enrichment analysis that estimates the level of enrichment for a specific biological process in a sample population in an unsupervised manner (20). We obtained the set of genes associated with anti-tumor or pro-tumor immunity (21), stromal activation (22, 23) and carcinogenic activation (24), summarized from previous studies, and calculated the activity of the corresponding biological processes by GSVA.

### Construction of the IRTF Score

To quantify the level of IRTF regulation in each patient, we devised a scoring system and evaluated the IRTF score. The process to determine the score was as follows:

We first identified differentially expressed genes (DEGs) with different regulation patterns in the combined cohort (|LogFC| > 1), and further selected genes with high discrimination (AUC > 0.8) between the two regulation patterns to ensure that the scoring system retained as much of the IRTF regulation characteristics as possible. Then, the univariate Cox regression and the KM method were used together to screen for the total survival-related genes; the genes with p-values < 0.01 in both the methods were included in the subsequent analysis. We then used the random forest method to further reduce the dimension based on the “src” function, parameter: ntree = 1000, seed = 12345678 and the rest of the parameters are default, then we selected the genes with importance > 0.2 as the final set of characteristic genes. Finally, multivariate Cox regression analysis was performed to generate the IRTF score for each patient. The receiver operating characteristic (ROC) analysis was used to determine whether the IRTF score retained the characteristics of the IRTF regulation patterns.

### Tumor Immune Dysfunction and Exclusion (TIDE) Analysis

TIDE (http://tide.dfci.harvard.edu) is an algorithm constructed by Jiang et al. to predict the response to immunotherapy, by primarily integrating the expression levels of T cell dysfunction and T cell exclusion. We predicted the response of immunotherapy, mainly to the anti-PD1 and anti-CTLA4 therapies (25). A TIDE score < 0 indicated that the patient was more likely to benefit from immunotherapy.

### Statistical Analysis

All statistical analyses and visualization were performed in the R software (version 3.60). The “limma” package was used to identify DEGs for the different regulation patterns. Pearson’s χ2 test or Fisher’s exact test was performed to compare the differences between categorical variables, and the Wilcoxon rank-sum test was performed to compare the differences between continuous variables. Spearman’s correlation test and the distance correlation analysis were performed to compare the correlations between continuous variables. The ROC curves were used to assess the specificity and sensitivity of the genes and IRTF scores using the “pROC” R package, as well as to find the best cut-off value. For survival analysis, The KM method and the log-rank test were used to compare the survival distribution; the cut-off point for each continuous variable was determined using the “survminer” package. Cox regression analysis was performed to identify the prognostic variables and calculate the β regression coefficient, hazard ratios (HR), p-value, and their corresponding 95% confidence intervals. All statistical tests were two-sided, and a p-value < 0.05 was considered to be statistically significant unless otherwise stated.

## Results

### Identification of IRTFs

**Figure 1:**
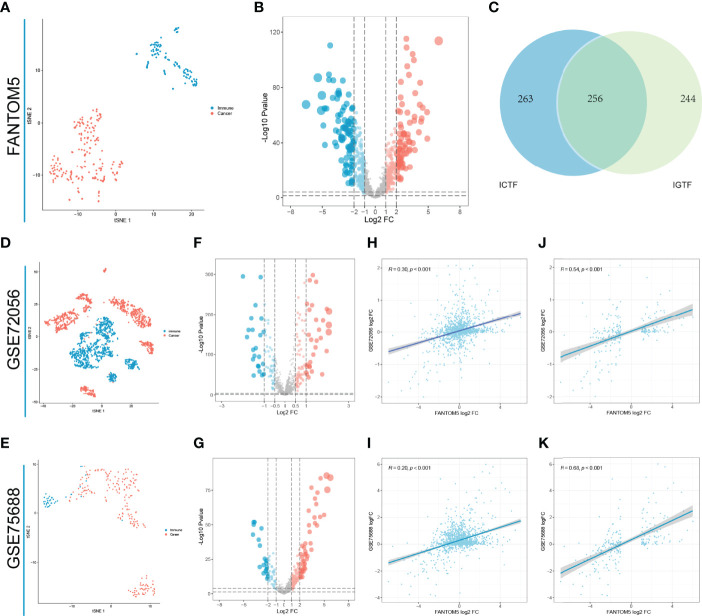
Identification of IRTFs. **(A)** The t-SNE result of classifying 110 immune cells and 194 solid cancer cell lines from FANTOM5. **(B)** The differential expression analysis between 110 immune cells and 194 cancer cell lines from FANTOM5. **(C)** Identification of 256 IRTFs. **(D, E)** The t-SNE results of classifying immune cells and 194 tumor cells from GSE72056 and GSE75688. **(F, G)** The differential expression analysis between immune cells and tumor cells from GSE72056 and GSE75688. **(H, I)** Expression patterns of TFs in GSE72056 (R = 0.30) and GSE75688 (R = 0.20) were significantly correlated with those in FANTOM5. **(J, K)** Expression patterns of IRTFs in GSE72056 (R = 0.54) and GSE75688 (R = 0.68) were more correlated with those in FANTOM5.

Based on ChEA3, we first performed regulation factor enrichment analysis for all immune-related genes by defining the TFs with the top 500 mean ranks as immune gene-related TFs (IGTFs). For FANTOM5, we found that immune cells and tumor cell lines had different expression patterns, based on the t-SNE analysis and the differential expression analysis (**Figures 1A, B**). Thus, we defined the differentially expressed TFs (|log2FC| > 1) as immune cell-associated TFs (ICTFs). Finally, we selected the 256 TFs screened in both the analyses mentioned above as the final defined IRTFs (**Figure 1C** and **Table 3**). Additionally, to validate the generality of the expression pattern of IRTFs, we repeated the above steps in two single-cell datasets (GSE75688, GSE72056) containing cancer cells and immune cells (**Figures 1D–G**) and compared the log2FC correlation of all TFs between FANTOM5 and the single-cell datasets. The results demonstrated a modest consistency between FANTOM5 and the single-cell datasets (**Figures 1H–J**). We then compared the log2FC correlation of IRTFs between FANTOM5 and the single-cell datasets. Interestingly, the results showed a higher consistency between FANTOM5 and the single-cell datasets (**Figures 1J, K**), indicating that IRTFs might play a critical and stable role in cancer immunity. Furthermore, the KEGG enrichment analysis showed that IRTFs were involved in important biological pathways, including cancers, the immune system, cell growth and death, and signal transduction (**Figure S1B** and **Table 4**).

**Table 3:** List of immune-related transcription factors (IRTF).

IRTF	ChEA3 Rank	FANTOM logFC
IRF8	1	-4.899
IRF5	2	-2.963
STAT4	3	-4.595
FOXP3	4	-1.850
BATF	5	-4.360
TBX21	6	-3.191
SP140	7	-5.657
PLSCR1	8	-1.285
TFEC	9	-3.655
IRF7	10	-3.223
NFKB2	12	-2.808
RELB	13	-2.135
IKZF1	14	-6.939
CSRNP1	15	-2.976
IRF1	16	-4.014
ARID5A	17	-4.254
SP110	18	-3.489
IRF9	19	-3.284
IKZF3	20	-3.794
STAT1	21	-1.224
STAT5A	22	-3.958
SPI1	23	-6.535
IRF4	24	-4.369
RUNX3	27	-5.040
POU2F2	28	-3.572
HLX	29	-1.696
MXD1	30	-3.110
SPIB	31	-1.487
MTF1	32	-1.784
ZNF267	33	-3.397
NFKB1	35	-3.589
SP140L	36	-2.480
SP100	37	-3.218
CEBPB	38	-1.385
STAT5B	39	-1.628
ELF4	40	-2.745
NFATC2	41	-1.539
TCF7	42	-1.898
GFI1	43	-1.569
MSC	44	-1.371
AKNA	46	-3.512
TRAFD1	48	-1.228
LTF	49	-1.217
BCL6	52	-2.794
ETV7	53	-1.369
PRDM1	54	-4.776
IRF2	55	-2.018
KLF2	57	-5.102
SCML4	58	-3.434
FLI1	59	-5.417
RFX5	60	-1.038
SNAI3	63	-2.692
EGR2	64	-2.388
ETS1	66	-2.194
NFE2	69	-1.871
EOMES	70	-1.107
KLF4	71	-1.056
USF1	72	-1.050
TET2	73	-2.947
ZNF467	75	-1.493
LYL1	77	-3.453
RARA	78	-2.235
NFIL3	79	-1.254
STAT3	80	-1.383
REL	81	-3.433
LEF1	83	-2.188
JUNB	84	-3.119
ZNF366	85	-1.066
STAT2	86	-1.386
CEBPA	87	-1.222
EPAS1	88	2.877
ATF5	91	1.273
BHLHE40	92	-1.470
MAF	95	-2.743
RUNX2	96	-1.401
MAFB	98	-2.537
AHR	99	-1.972
PPARD	102	-1.243
TFEB	104	-1.929
NR3C1	105	-2.353
FOS	107	-2.203
HIC1	109	-1.550
PROX1	113	1.662
DDIT3	114	-1.094
HHEX	115	-1.228
FOSB	117	-2.193
STAT6	119	-2.117
RUNX1	122	-2.191
SOX18	123	1.056
ASCL2	125	-1.819
ETV3	127	-1.737
EGR1	128	1.060
FOSL1	129	1.679
ESR1	132	-1.080
MSX1	135	2.357
ZNF641	137	-1.086
NFATC1	138	-3.058
BACH1	140	-2.345
VDR	141	-1.702
MAX	143	-1.067
ETV6	144	-1.749
HES1	145	2.410
GATA3	146	-1.102
EGR3	150	-1.354
FOXO1	152	-2.450
BCL11B	155	-2.860
KLF5	156	2.164
NR4A3	158	-2.648
TBX2	159	2.088
SOX7	160	1.209
ARNTL	161	-2.483
PBX4	164	-1.287
ZNF438	166	-2.618
FOSL2	167	-1.817
FOXA2	168	1.406
TWIST1	171	2.488
ZNF394	172	-2.197
AR	173	1.130
FOXC2	177	1.343
GATA2	178	2.499
FOXP2	182	1.026
KLF6	183	-2.899
RORA	184	-1.615
ZNF350	185	-2.335
GTF2B	186	-1.776
ELK3	187	-1.557
NR2F2	189	5.996
TFAP2C	191	2.585
TIGD2	196	1.093
PRRX1	198	1.296
ZNF746	200	-1.019
ELF1	202	-3.059
ZNF331	203	-2.267
GTF2I	204	1.538
FOXQ1	205	2.388
SNAI2	206	3.742
ZNF217	207	-1.049
TBX3	209	3.264
NFE2L3	211	1.191
NR4A2	212	-3.199
SOX9	214	4.122
PRRX2	221	1.402
GATA6	225	3.185
OSR2	227	1.100
ZEB1	228	-1.126
PCGF2	230	5.097
ZEB2	233	-4.751
GATA4	236	1.430
HOXA9	238	1.280
TWIST2	240	1.314
SP6	241	1.205
KLF3	245	-1.836
RARB	246	1.413
CREM	251	-3.082
SREBF1	252	1.414
ZBTB49	253	-1.356
ZNF101	254	-2.036
EHF	257	1.445
HES2	258	1.164
ZBTB17	259	-1.298
HOXB7	261	2.852
ELF3	264	3.279
MEIS1	265	2.295
TFAP2A	266	4.898
HOXA3	268	2.230
TEAD3	270	3.651
ZNF586	271	-1.501
ZNF75D	272	-1.015
FOXA1	274	2.160
NR1H2	275	-1.434
ATOH8	276	1.348
MEF2A	277	-1.099
SMAD1	282	2.287
BACH2	284	-2.763
MYC	285	1.533
JUND	287	-2.004
RBPJ	289	-1.069
ZHX2	293	-2.399
NRF1	294	-1.173
NCOA3	296	-1.150
ZNF276	297	-2.507
TEAD1	298	5.463
FOXL2	303	1.007
SP5	308	1.051
IRF6	310	1.705
DLX3	313	1.520
CREB1	314	-1.489
SMAD5	316	1.854
PHF21A	317	-1.123
MLXIPL	318	1.466
SATB1	319	-3.725
NFATC3	326	-1.482
KLF9	327	-1.422
ARNTL2	332	2.493
IRX2	333	1.829
TBX18	334	1.378
ARID3B	337	-1.158
NCOA2	338	-1.919
GRHL2	341	1.653
TRPS1	345	-1.857
BNC1	346	1.331
SALL1	347	1.005
POU2AF1	350	-1.497
OVOL1	355	1.085
KLF13	361	-2.548
NPAS2	362	3.218
TP73	364	1.360
SIX5	367	3.024
HBP1	369	-2.138
ZBTB1	371	-1.926
ZNF683	378	-1.257
MSX2	380	2.230
ZNF462	382	2.445
MBD2	385	-1.387
ZBTB48	387	-1.152
PBX1	388	3.551
GTF2IRD1	391	3.534
SOX2	392	1.773
SOX13	393	2.552
ONECUT2	397	2.264
IRX3	398	2.830
MITF	399	1.147
ISL1	402	1.191
MBD4	406	-1.164
GRHL1	408	1.207
MECOM	409	2.299
IRX5	411	1.120
GRHL3	414	1.024
PAX9	417	1.742
ZBTB32	421	-1.192
TCF7L1	425	1.302
KLF7	428	-2.194
TEAD4	429	4.707
GLI3	431	1.803
MEIS2	436	3.775
SOX15	438	1.064
NR2F6	439	3.185
SMAD9	441	1.688
FOXP1	442	-1.915
FOXO3	443	-1.248
ZNF117	446	-1.123
FOXN2	447	-1.734
SCMH1	448	1.048
HMGA2	454	4.920
PLAGL1	457	-2.174
HES4	462	1.273
HIVEP1	463	-2.164
CREB3L1	464	2.646
L3MBTL3	465	-1.526
BNC2	476	1.386
ZBED3	480	1.455
ZBTB7A	481	-1.451
MEF2C	490	-2.843
PAX6	492	2.066
E2F5	494	1.574
EBF4	498	1.022

**Table 4:** KEGG enrichment analysis of IRTFs.

Term	Description	Adj p-value	Count
hsa05202	Transcriptional misregulation in cancer	0.000	28
hsa05203	Viral carcinogenesis	0.000	15
hsa05235	PD-L1 expression and PD-1 checkpoint pathway in cancer	0.001	8
hsa05221	Acute myeloid leukemia	0.000	12
hsa05215	Prostate cancer	0.001	8
hsa05224	Breast cancer	0.004	9
hsa05213	Endometrial cancer	0.012	5
hsa05216	Thyroid cancer	0.014	4
hsa05223	Non-small cell lung cancer	0.025	5
hsa05220	Chronic myeloid leukemia	0.030	5
hsa05210	Colorectal cancer	0.046	5
hsa04218	Cellular senescence	0.002	10
hsa04217	Necroptosis	0.019	8
hsa04933	AGE-RAGE signaling pathway in diabetic complications	0.002	8
hsa04931	Insulin resistance	0.003	8
hsa04950	Maturity onset diabetes of the young	0.004	4
hsa04934	Cushing syndrome	0.046	7
hsa04928	Parathyroid hormone synthesis, secretion and action	0.000	12
hsa04917	Prolactin signaling pathway	0.000	9
hsa04935	Growth hormone synthesis, secretion and action	0.004	8
hsa04919	Thyroid hormone signaling pathway	0.016	7
hsa04916	Melanogenesis	0.025	6
hsa04915	Estrogen signaling pathway	0.029	7
hsa04659	Th17 cell differentiation	0.000	17
hsa04658	Th1 and Th2 cell differentiation	0.000	15
hsa04625	C-type lectin receptor signaling pathway	0.000	12
hsa04657	IL-17 signaling pathway	0.019	6
hsa04662	B cell receptor signaling pathway	0.039	5
hsa05161	Hepatitis B	0.000	18
hsa05166	Human T-cell leukemia virus 1 infection	0.000	19
hsa05167	Kaposi sarcoma-associated herpesvirus infection	0.000	15
hsa05162	Measles	0.001	10
hsa05169	Epstein-Barr virus infection	0.001	12
hsa05165	Human papillomavirus infection	0.006	14
hsa05160	Hepatitis C	0.048	7
hsa04390	Hippo signaling pathway	0.001	11
hsa04022	cGMP-PKG signaling pathway	0.003	10
hsa04310	Wnt signaling pathway	0.007	9
hsa04630	JAK-STAT signaling pathway	0.007	9
hsa04668	TNF signaling pathway	0.012	7
hsa04010	MAPK signaling pathway	0.014	12
hsa04392	Hippo signaling pathway - multiple species	0.040	3

### IRTF Regulation Patterns Mediated by IRTFs

**Figure 2:**
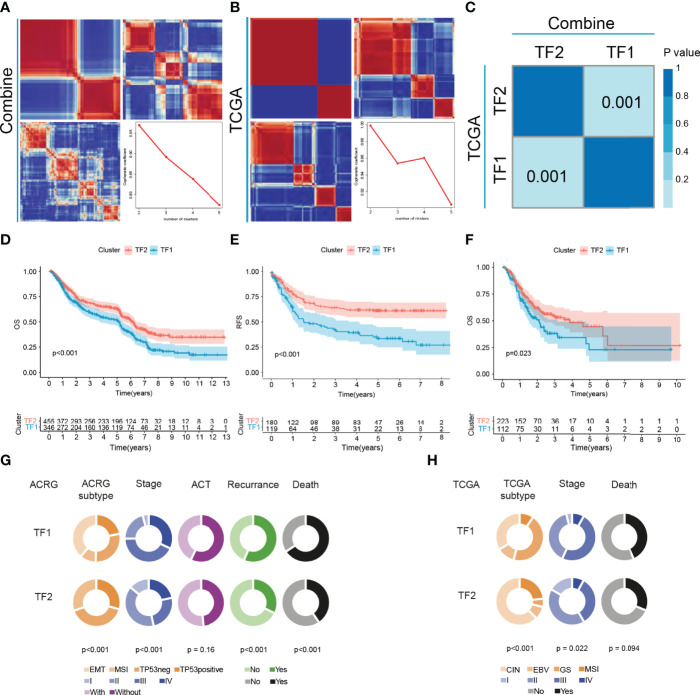
The landscape of clinical features of IRTFs. **(A, B)** Consensus matrix of the NMF clustering and cophenetic correlation coefficient along with the corresponding k values for the combined cohort **(A)** and the TCGA-STAD cohort **(B)**. **(C)** SubMap analysis showed a significant correlation between the combined cohort and the TCGA-STAD cohort. **(D–F)** OS and RFS analyses of the two IRTF regulation patterns for the combined cohort **(D, E)** and the TCGA-STAD cohort **(F)**. **(G, H)** The alluvial diagram showing the correlation between IRTF regulation patterns and clinical phenotypes for the ACRG cohort **(G)** and the TCGA-STAD cohort **(H)**.

Five gastric cancer datasets (GSE15459, GSE34942, GSE57303, GSE62254/ACRG and PUCH) with available OS data and clinical information were enrolled into combined cohort. In this cohort, 251 of the 256 IRTFs were identified. Next, NMF regulation patterning was conducted on a combined cohort using 251 IRTFs to identify distinct IRTF regulation patterns; cophenetic correlation coefficients were used to select the optimal number of regulation patterns. We identified two distinct regulation patterns (defined as TF1 and TF2) (**Figure 2A**). Subsequently, the same procedures were performed in an independent TCGA-STAD cohort, and two regulation patterns were identified (**Figure 2B**). SubMap analysis of full gene expression profiles showed that TF1 and TF2 in the combined cohort were highly correlated with the corresponding classification in the TCGA-STAD cohort, indicating the robustness and consistency of the classification (**Figure 2C**). A prognostic analysis showed a significant survival and recurrence-free advantages of the TF2 over the TF1 in both cohorts (**Figures 2D–F**). To further explore the characteristics of these IRTF regulation patterns in important clinical phenotypes, we focused on the ACRG cohort. Patients with stage II III GC are considered to be the group most likely to benefit from surgery or adjuvant chemotherapy (26); prognostic analysis of stage II III GC showed a particularly prominent OS and RFS advantage in TF2 (**Figures S2A, B**). We further performed the prognostic analysis in groups receiving and not receiving adjuvant chemotherapy, and showed that the OS and RFS advantages in TF2 were independent of the effect of chemotherapy on the prognosis of GC (**Figures S2C–F**). Additionally, pie charts showed the correlation of IRTF regulation patterns with important clinical phenotypes; TF2 focused on the earlier clinical stages, the MSI subtypes (ACRG and TCGA subtypes), and was associated with lower mortality and recurrence rates, while TF1 focused on the higher clinical stages, the EMT (ACRG subtypes) and GS subtypes (TCGA subtypes), and was associated with higher mortality and recurrence rates (**Figures 2G, H**).

### Immune Characteristics in Distinct IRTF Regulation Patterns

**Figure 3:**
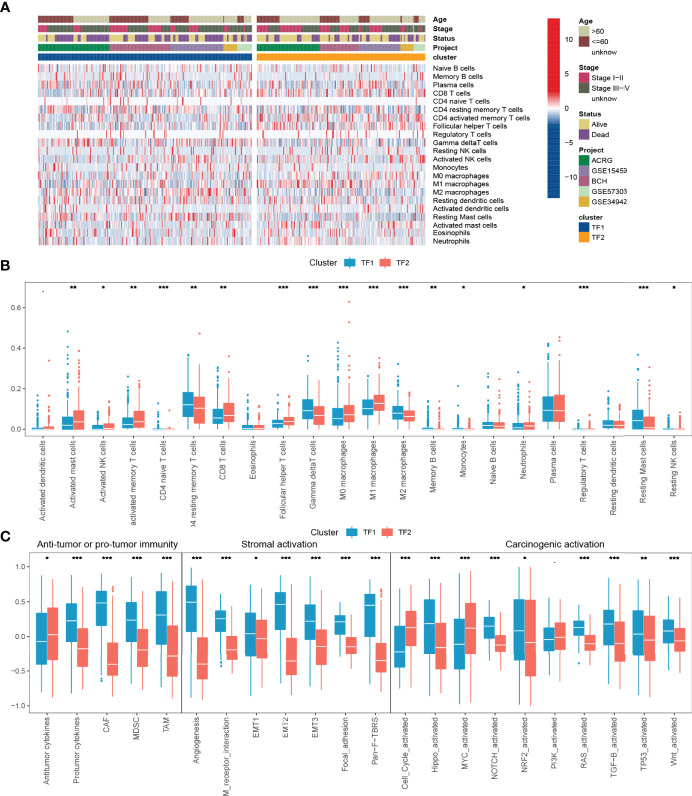
TIME characteristics and transcriptome traits for distinct IRTF regulation patterns. **(A)** The distribution characteristics of 22 immune cells and clinical phenotypes for distinct IRTF regulation patterns. **(B)** A comparative analysis of the differences between 22 immune cell types for distinct IRTF regulation patterns. **(C)** The GSVA analysis showed differences in tumor immunity, stroma-activated pathways and common carcinogenic pathways. ***: < 0.001; **: < 0.01; *: < 0.05.

To further characterize and understand the TIME of GC, we focused on the combined cohort. We compared the differences in immune infiltration between the two regulation patterns based on CIBERSORT and showed that TF2 was characterized by an increase in the infiltration of anti-tumor immune cells, such as CD8 T cells, M1 macrophages, and NK cells, and a decrease in the infiltration of tumor-promoting immune cells, such as M2 macrophages (**Figures 3A, B**). Subsequent another analyses revealed an overall decrease of cancer-associated fibroblasts (CAFs), tumor-associated Macrophages (TAMs) and Myeloid-derived suppressor cells (MDSC), and protumor cytokines in TF2 and an increase of Antitumor cytokines in TF2 (**Figure 3C**, **Table 5**). TF2 was therefore classified as an immune-activating phenotype. Additionally, stromal activation in TIME is thought to suppress T cell function (27). GSVA analysis showed that TF1 correlated significantly with stroma-activated pathways (**Figure 3C** and **Table 5**). Therefore, we hypothesized that stromal activation of TF1 would inhibit the anti-tumor effects of immune cells. We then referred to published signatures of common carcinogenic pathways (**Table 5**). The Hippo, NOTCH, NRF2, RAS, TGF-B, TP53, and wnt pathways had higher scores in TF1, and only MYC and cell cycle pathways had higher scores in TF2. Overall, TF1 exhibited a relatively pronounced phenotype of oncogenic activation (**Figure 3D**). We, therefore, classified TF1 as a stromal and oncogenic activation phenotype.

**Table 5:** Gene signatures enrolled in this study.

Gene signature	Genes	Source
Antitumor cytokines	TNF, IFNB1, IFNA2, CCL3, TNFSF10, IL21	PMID: 34019806
Protumor cytokines	IL10, TGFB1, TGFB2, TGFB3, IL22, MIF, IL6	
MDSC	CSF2, CSF3, CXCL12, CCL26, IL6, CXCL8, CXCL5, CSF1R, CSF2RA, CSF3R, CXCR4, IL6R, CXCR2, CCL15, CSF1	
TAM	IL10, MRC1, MSR1, CD163, CSF1R, IL4I1, SIGLEC1, CD68	
CAF	COL1A1, COL1A2, COL5A1, ACTA2, FGF2, FAP, LRP1, CD248, COL6A1, COL6A2, COL6A3, CXCL12, FBLN1, LUM, MFAP5, MMP3, MMP2, PDGFRB, PDGFRA	

EMT1	CLDN3, CLDN7, CLDN4, CDH1, VIM, TWIST1, ZEB1, ZEB2	PMID: 24520177
EMT2	AXL, FAP, LOXL2, ROR2, TAGLN, TWIST2, WNT5A	PMID: 26997480
EMT3	FOXF1, GATA6, SOX9, TWIST1, ZEB1, ZEB2	PMID: 27321955
Pan-F-TBRS	ACTA2, ACTG2, ADAM12, ADAM19, CNN1, COL4A1, CTGF, CTPS1, FAM101B, FSTL3, HSPB1, IGFBP3, PXDC1, SEMA7A, SH3PXD2A, TAGLN, TGFBI, TNS1, TPM1	PMID: 29443960
Angiogenesis	CDH5, SOX17, SOX18, TEK	PMID: 22553347
ECM_RECEPTOR_INTERACTION	GP1BA, COL6A2, COL6A3, GP1BB, COL5A2, COL6A1, LAMA1, VWF, HSPG2, TNN, FN1, ITGA9, GP9, COMP, IBSP, CD36, CHAD, GP5, VTN, THBS4, ITGA4, ITGA3, ITGA2B, ITGA7, ITGA5, COL5A1, COL4A6, ITGA11, SV2C, COL2A1, COL3A1, COL4A1, AGRN, COL4A2, COL4A4, ITGB3, ITGB4, RELN, ITGB5, ITGB6, ITGB7, LAMC2, ITGAV, ITGB1, LAMB2, SPP1, LAMB3, LAMC1, COL1A1, LAMA4, LAMA5, LAMB1, COL1A2, ITGA10, GP6, ITGA8, LAMB4, TNR, CD47, SV2A, CD44, DAG1, TNXB, LAMA3, LAMA2, SDC3, ITGB8, ITGA6, ITGA2, ITGA1, SV2B, TNC, COL11A1, LAMC3, COL11A2, HMMR, SDC2, SDC4, COL5A3, THBS3, COL6A6, THBS2, SDC1, THBS1	KEGG: map04512
FOCAL_ADHESION	JUN, ELK1, HGF, PARVA, FN1, TNN, IGF1, BIRC3, XIAP, COMP, THBS4, IGF1R, DIAPH1, ITGA11, PGF, PARVG, ROCK1, PTK2, MYL7, FLT1, FLT4, AKT1, RELN, AKT2, LAMC2, MYL12A, LAMB2, LAMB3, LAMC1, LAMA4, LAMA5, LAMB1, PAK6, PIK3R5, CAPN2, LAMB4, FLNC, FLNA, FLNB, MYL2, MYLK, MYL5, PIP5K1C, MET, MYL10, BIRC2, COL11A1, LAMC3, COL11A2, THBS3, THBS2, THBS1, VWF, ZYX, IBSP, VTN, PDGFD, PPP1R12A, BAD, ACTN4, ACTN1, MAPK9, MAPK10, MAP2K1, RASGRF1, ILK, RAPGEF1, GRB2, PPP1CC, PPP1CB, ACTG1, ITGA10, HRAS, ITGA8, CTNNB1, MYL12B, ACTB, ROCK2, PTEN, RAP1A, PIK3R3, RAP1B, TNC, CAV2, CAV1, CAV3, COL5A3, TLN1, VAV3, COL6A2, COL6A3, COL5A2, COL6A1, LAMA1, ITGA9, CHAD, PAK4, ITGA4, ITGA3, ITGA2B, ITGA7, ITGA5, COL5A1, COL4A6, PDGFRB, COL2A1, COL3A1, COL4A1, PARVB, COL4A2, BRAF, COL4A4, VAV1, PDPK1, ITGB3, ITGB4, VASP, ITGB5, SHC4, ITGB6, DOCK1, ITGB7, ITGAV, ITGB1, AKT3, VAV2, SPP1, COL1A1, COL1A2, TLN2, PDGFC, VCL, SHC3, VEGFA, VEGFC, ITGB8, VEGFB, PXN, PAK5, CCND1, PDGFA, BCL2, PDGFB, PDGFRA, ARHGAP5, BCAR1, PAK1, VEGFD, CRK, CRKL, CCND2, CDC42, ACTN2, CCND3, ACTN3, SOS2, PAK3, PRKCB, RAF1, PRKCA, SHC1, PAK2, MYL9, RHOA, PRKCG, MYLPF, ERBB2, RAC2, RAC3, KDR, MYLK2, PPP1CA, MAPK3, ARHGAP35, RAC1, SOS1, MAPK1, MAPK8, EGFR, GSK3B, TNR, EGF, LAMA3, TNXB, LAMA2, ITGA6, ITGA2, SRC, ITGA1, PIK3CA, PIK3CB, PIK3CD, SHC2, COL6A6, MYLK3, FYN, PIK3CG, PIK3R1, PIK3R2	KEGG: map04510

Cell_Cycle_activated	CCND1, CCND2, CCND3, CCNE1, CDK2, CDK4, CDK6, E2F1, E2F3	PMID: 29625050
Hippo_activated	YAP1, TEAD1, TEAD2, TEAD3, TEAD4, WWTR1	
MYC_activated	MYC, MYCL1, MYCN	
NOTCH_activated	CREBBP, EP300, HES1, HES2, HES3, HES4, HES5, HEY1, HEY2, HEYL, KAT2B, NOTCH1, NOTCH2, NOTCH3, NOTCH4, PSEN2, LFNG, NCSTN, JAG1, APH1A, FHL1, THBS2, MFAP2, RFNG, MFAP5, JAG2, MAML3, MFNG, CNTN1, MAML1, MAML2, PSEN1, PSENEN, RBPJ, RBPJL, SNW1, ADAM10, APH1B, ADAM17, DLK1, DLL1, DLL3, DLL4, DNER, DTX1, DTX2, DTX3, DTX3L, DTX4, EGFL7	
NRF2_activated	NFE2L2	
PI3K_activated	EIF4EBP1, AKT1, AKT2, AKT3, AKT1S1, INPP4B, MAPKAP1, MLST8, MTOR, PDK1, PIK3CA, PIK3CB, PIK3R2, RHEB, RICTOR, RPTOR, RPS6, RPS6KB1, STK11,	
TGF-B_activated	TGFBR1, TGFBR2, ACVR2A, ACVR1B, SMAD2, SMAD3, SMAD4	
TP53_activated	TP53, ATM, CHEK2, RPS6KA3	
Wnt_activated	LEF1, LGR4, LGR5, LZTR1, NDP, PORCN, SFRP1, SFRP2, SFRP4, SFRP5, SOST, TCF7L1, WIF1, ZNRF3, CTNNB1, DVL1, DVL2, DVL3, FRAT1, FRAT2, DKK1, DKK2, DKK3, DKK4, RNF43, TCF7, TCF7L2	
RAS_activated	ABL1, EGFR, ERBB2, ERBB3, ERBB4, PDGFRA, PDGFRB, MET, FGFR1, FGFR2, FGFR3, FGFR4, FLT3, ALK, RET, ROS1, KIT, IGF1R, NTRK1, NTRK2, NTRK3, SOS1, GRB2, PTPN11, KRAS, HRAS, NRAS, RIT1, ARAF, BRAF, RAF1, RAC1, MAP2K1, MAP2K2, MAPK1, INSR, INSRR, IRS1, SOS2, SHC1, SHC2, SHC3, SHC4, RASGRP1, RASGRP2, RASGRP3, RASGRP4, RAPGEF1, RAPGEF2, RASGRF1, RASGRF2, FNTA, FNTB, SPRED1, SPRED2, SPRED3, SHOC2, KSR1, KSR2, JAK2, IRS2	

We further investigated the differences in immunogenicity between distinct IRTF regulation patterns. We first assessed the distribution of the somatic variants of GC driver genes in TF1 and TF2. The top 20 genes in terms of mutation frequency were analyzed and visualized (**Figure S3A**), and the results showed significant differences in the mutation frequencies of TTN, TP53, LRP1B, DNAH5, CSMD1, SYNE1, ZFHX4, OBSCN, and FAT4 (chi-squared test; **Table 6**) between TF1 and TF2. Accumulation of driver mutations might lead to higher immunogenicity in the TF2. Additionally, Thorsson et al. studied the pan-cancer immune landscape in TCGA and calculated several indicators that affect the immunogenicity of tumors, including tumor mutation burden (TMB), single nucleotide variant (SNV) neoantigens, intratumor heterogeneity (ITH), cancer-testis antigen (CTA) score, homologous recombination deficiency (HRD), aneuploidy score, CNV burden (number of segments and fraction of genome alterations), loss of heterozygosity (number of segments with LOH events and the fraction of bases with LOH events) (28). We compared the differences in immunogenicity between the two regulation patterns and found that TF2 had relatively higher TMB, SNV neoantigens, ITH, CTA score, HRD, aneuploidy score, CNV burden, and loss of heterozygosity. Additionally, the two regulation patterns had no significant differences in ITH (**Figures S3B–K**).

**Table 6:** Differential mutation analysis of the top 20 genes.

Gene	TF1 wild	TF1 mutation	TF2 wild	TF2 mutation	P-value
TTN	73 (69.52%)	32 (30.48%)	101 (45.5%)	121 (54.5%)	0.000
TP53	77 (73.33%)	28 (26.67%)	113 (50.9%)	109 (49.1%)	0.000
LRP1B	91 (86.67%)	14 (13.33%)	156 (70.27%)	66 (29.73%)	0.002
DNAH5	98 (93.33%)	7 (6.67%)	178 (80.18%)	44 (19.82%)	0.004
CSMD1	98 (93.33%)	7 (6.67%)	180 (81.08%)	42 (18.92%)	0.006
SYNE1	91 (86.67%)	14 (13.33%)	164 (73.87%)	58 (26.13%)	0.014
ZFHX4	97 (92.38%)	8 (7.62%)	182 (81.98%)	40 (18.02%)	0.021
OBSCN	97 (92.38%)	8 (7.62%)	182 (81.98%)	40 (18.02%)	0.021
FAT4	93 (88.57%)	12 (11.43%)	173 (77.93%)	49 (22.07%)	0.031
HMCN1	95 (90.48%)	10 (9.52%)	181 (81.53%)	41 (18.47%)	0.055
KMT2D	95 (90.48%)	10 (9.52%)	182 (81.98%)	40 (18.02%)	0.068
CSMD3	91 (86.67%)	14 (13.33%)	174 (78.38%)	48 (21.62%)	0.102
RYR2	95 (90.48%)	10 (9.52%)	186 (83.78%)	36 (16.22%)	0.146
FLG	90 (85.71%)	15 (14.29%)	175 (78.83%)	47 (21.17%)	0.183
PCLO	92 (87.62%)	13 (12.38%)	180 (81.08%)	42 (18.92%)	0.188
MUC16	79 (75.24%)	26 (24.76%)	150 (67.57%)	72 (32.43%)	0.199
SPTA1	93 (88.57%)	12 (11.43%)	186 (83.78%)	36 (16.22%)	0.330
FAT3	93 (88.57%)	12 (11.43%)	188 (84.68%)	34 (15.32%)	0.439
ARID1A	79 (75.24%)	26 (24.76%)	172 (77.48%)	50 (22.52%)	0.759
PIK3CA	91 (86.67%)	14 (13.33%)	190 (85.59%)	32 (14.41%)	0.927

### Construction and Evaluation of the IRTF Score System

The above results suggested that IRTF regulation patterns play a significant role in the treatment and prognosis of GC; however, the analyses in those studies were conducted at a population scale, and thus, cannot accurately predict the IRTF regulation patterns in individual patients. We, therefore, constructed the IRTF score system and characterized the expression patterns of the signature genes (**Figures S4A–D**). The ROC analysis showed that the IRTF score system could distinguish the two regulation patterns well (**Figures S4E, F**), indicating that the characteristics of the IRTF regulation patterns were well preserved.

We then assessed the correlation of the IRTF scores with OS and RFS; the results showed that lower IRTF scores indicated a significant prognostic advantage (**Figures S5A–D**). Next, we analyzed the correlation between the IRTF scores and clinical phenotypes and found that the IRTF scores were significantly correlated with survival status, age, stage, T stage, M stage, and MSI (**Figure S5E**). We determined whether the IRTF score could serve as an independent prognostic biomarker. Multivariate Cox regression analysis of the TCGA-STAD and ACRG cohorts confirmed that the IRTF score was a robust and independent prognostic biomarker for evaluating patient prognosis (**Figures S5F, G**).

**Figure 4:**
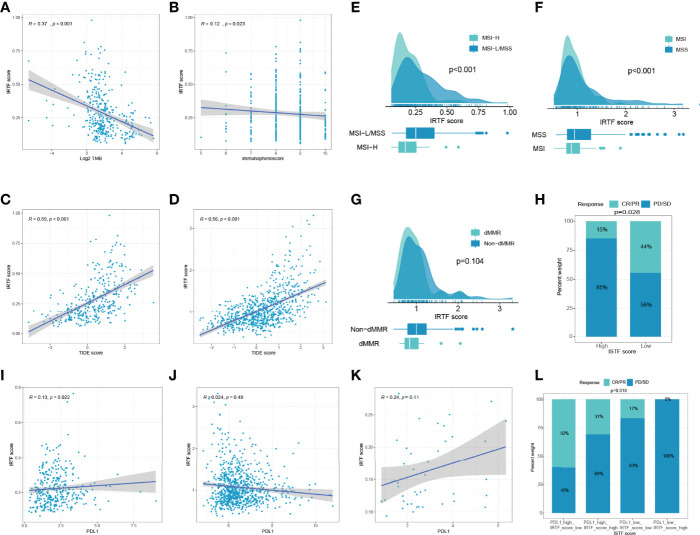
IRTF score system in the role of immunotherapy of GC. **(A–D)** Correlation analysis of IRTF scores with immunotherapy-related markers, including TMB, immunophenoscore, and TIDE scores for the TCGA-STAD cohort **(A–C)** and the combined cohort **(D)**. Correlation analysis of IRTF scores with MSI/dMMR status for the TCGA-STAD cohort **(E)**, the ACRG cohort **(F)**, and the PUCH cohort **(G)**. **(H)** The proportion of GC patients showing response to anti-PD1 antibody in the low or high IRTF score groups. **(I–K)** Correlation analysis of IRTF scores with PDL1 for the TCGA-STAD cohort **(I)**, the combined cohort **(J)**, and the ERP107734 cohort **(K)**. **(L)** The proportion of GC patients showing response to anti-PD1 antibody in four different groups based on the PDL1 mRNA and the IRTF scores.

We then evaluated the feasibility of the IRTF score as a marker for immunotherapy. A scatter plot and Spearman’s correlation analysis showed that the IRTF score was negatively correlated with TMB and immunophenoscore, and significantly positively correlated with the TIDE score (**Figures 4A–D**). Further analysis also showed that the IRTF score was higher in the MSI-H/MSI/dMMR subgroup than in the MSS/MSS/MMR subgroup (**Figures 4E–G**). Finally, we evaluated the efficacy of response to anti-PD1 antibody in the high and low IRTF score subgroups (best cut-off value) of the ERP107734 cohort and were surprised to find that the response rate was significantly higher in the low IRTF score subgroup than in the high IRTF score subgroup (**Figure 4H**). Kim et al. had found that the PDL1 mRNA expression could be used as a marker to predict the efficacy of the PD1 antibody. However, the IRTF score was not found to correlate significantly with PDL1 mRNA expression (**Figures 4I–K**). Therefore, we speculated that the IRTF score might play a predictive role in immunotherapy independently of PDL1, and combining the two might optimize the prediction of the immunotherapy response. We divided the GC patients of the ERP107734 cohort into four groups based on the median IRTF score and PDL1 expression. Surprisingly, the low PDL1 and high IRTF score group showed a response rate of 0, while the high PDL1 and low IRTF score group showed a response rate of up to 60%. Additionally, the other two groups also obtained different response rates (**Figure 4L**).

### Multi-Cancer IRTF Score Analysis

First, we applied the IRTF scoring method to five digestive system cancers, including READ, COAD, PAAD, CHOL, and LIHC. Survival analysis showed that a low IRTF score was considered a favorable prognostic biomarker in five independent TCGA cohorts (**Figures S6A–E**), four of which were further confirmed in either the GEO or ICGC cohorts (**Figures S6F–I**). We did not find a validation cohort for CHOL.

**Figure 5:**
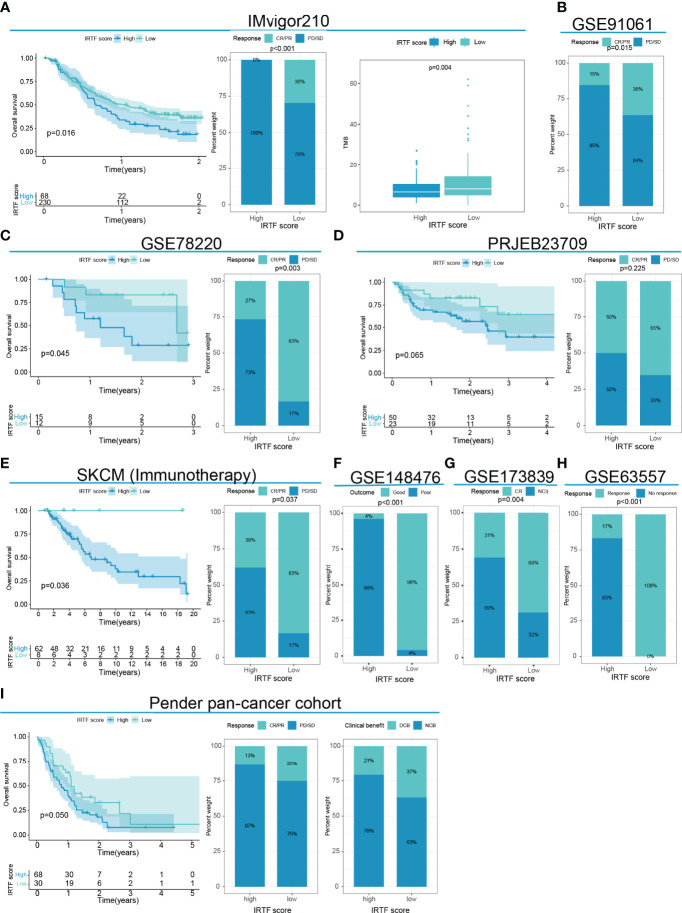
IRTF score system in the role of immunotherapy of multi-cancer. **(A)** Survival analysis, the proportion of patients showing a response, and TMB level in the low or high IRTF score groups of the IMvigor210 cohort with the anti-PDL1 antibody. **(B)** The proportion of patients showing a response to anti-PD1 antibody in the low or high IRTF score groups of the GSE91061 cohort. **(C)** Survival analysis and the proportion of patients showing a response in the low or high IRTF score groups of the GSE78220 cohort with anti-PD1 antibody. **(D)** Survival analysis and proportion of patients showing a response in the low or high IRTF score groups of the PRJEB23709 cohort with anti-PD1 antibody/anti-PD1 antibody+anti-CTLA4 antibody. **(E)** Survival analysis and proportion of patients showing a response in the low or high IRTF score groups of the SKCM (Immunotherapy) cohort with various types of immunotherapies. **(F)** The proportion of patient showing a response in the low or high IRTF score groups of the GSE148476 cohort with various types of immunotherapies. **(G)** The proportion of patient showing a response in the low or high IRTF score groups of the GSE173839 cohort with neoadjuvant immunotherapy **(H)** The proportion of mice showing a response to anti-CTLA4 antibody in the low or high IRTF score groups of the GSE63557 cohort. **(I)** Survival analysis and the proportion of patients showing a response and clinical benefit in the low or high IRTF score groups of the Pender pan -cancer immunotherapy cohort with various types of immunotherapies.

Next, we determined the value of the IRTF score for predicting the outcome of immunotherapy by dividing patients in six cohorts receiving immunotherapy into high or low IRTF score groups. For the IMvigor210 and GSE78220 cohorts, patients with lower IRTF scores had significantly higher survival and outcome benefits, as well as a higher TMB (**Figures 5A, C**). For the PRJEB23709 cohort, although the results obtained in the survival analysis and efficacy comparison were not statistically significant, a trend toward the benefit of immunotherapy was found in patients with lower IRTF scores (**Figure 5D**). Additionally, the predictive value of the IRTF score in immunotherapy was also confirmed for the SKCM (Immunotherapy) cohort (**Figure 5E**). Patients of the SKCM (Immunotherapy) cohort received various types of immunotherapies, including vaccines, cytokines, and checkpoint blockers. The results reflected the predictive value of the IRTF score in various types of immunotherapies. For the GSE91061, GSE148476 cohorts, as no survival information was available, we only compared the differences in efficacy between the high and low IRTF score groups (best cut-off value) and found an efficacy benefit for the low IRTF score group (**Figures 5B, F**). Interestingly, IRTF score was also found to be used as a biomarker for predicting efficacy in the GSE173839 cohort receiving neoadjuvant immunotherapy (**Figure 5G**). Next, we validated the predictive value of the IRTF score for the GSE63557 efficacy, a mouse model cohort treated with CTLA-4 antibody. The signature gene used to construct the IRTF score was transformed from mouse probes. Subsequently, we found that the immunotherapy efficacy was good in the low IRTF score subgroup (**Figure 5H**). In summary, the results of our multi-cohort analysis strongly suggested that the IRTF score system was correlated with the response to different immunotherapies.

## Discussion

This study included identification of IRTFs in determining the clinical and immunological characteristics of IRTF regulation patterns and construct the IRTF score system.

Based on 251 IRTFs, we identified two regulation patterns of GC with distinctly different immune profiles. In TIME, TF2 exhibited significant ‘hot tumor’ characteristics, inferred from the high infiltration of CD8 T cells, M1 macrophages, and NK cells, which are considered to be pro-inflammatory and anti-tumor immune cells (29, 30). We also found that Tregs were upregulated in TF2. Tregs promote tumor progression and dissemination by exerting immunosuppressive effects in tumors, thereby reducing the anti-tumor immune response (31). However, Tregs may also be associated with a good prognosis in GC, especially in patients with tumor location in the cardia or with MSI (32–34). Hence, the role of Tregs in GC remains controversial. TF1 not only exhibits “cold tumor” qualities, as shown by the downregulation of anti-tumor cells and upregulation of M2 macrophages but also exhibits stromal and oncogenic activation, which are thought to suppress T cells and increase tumor malignancy, respectively (24, 27). Hence, it was not surprising to find differences in survival between TF2 and TF1. Additionally, we identified 13 differentially infiltrating immune cells, none of which could be categorized as anti-GC or pro-GC; however, they differed significantly between the two modes (‘hot tumor’ and ‘cold tumor’) of presence in TIME. Regarding immunogenicity, there were significant differences between the two models in TMB, SNV neoantigens, CTA score, HRD, aneuploidy score, CNV burden, and loss of heterozygosity. TMB has been identified as a strong predictive marker for checkpoint blockers (35), and tumor neoantigens are considered important targets for cancer vaccines (36). We did not find a correlation between IRTF modulation patterns and ITH; however, recent studies have suggested that low ITH might be associated with better immunotherapy efficacy (37), indicating that IRTF regulation patterns play a role in predicting immunotherapy independent of ITH.

Immunotherapy, especially by checkpoint blockers, has made a positive impact on cancer treatment (38–40). Despite this, the responsiveness to immunotherapy shows interindividual variation. Therefore, it is of great clinical interest to find markers for predicting immunotherapy, especially those with multi-tumor predictive efficacy. Thus, we constructed a scoring system that can be used as a valid prognostic indicator not only in multiple digestive cancer cohorts receiving conventional treatment but also as a predictor of responses in multiple cancer cohorts receiving immunotherapies. More importantly, the combined prediction of the IRTF score and PDL1 established a detailed stratification of the response to the PD1 antibody in GC patients.

However, there were some limitations of our study. First, due to the algorithmic limitations, only 22 immune cells were evaluated, and the role of some tumor cells in GC tumor immunity was unclear. Second, for the neoadjuvant therapy in GC, a low sample size prevented better analysis. Finally, this study lacks a larger prospective study to further validate the findings.

## Conclusion

In this study, we provided new perspectives on tumor immunization and individualized therapy in GC. Evaluation of the IRTF modulation patterns in individual patients will help to enhance our understanding of immune specificities, and thus, guide rational and personalized therapeutic strategies.

In this study, we provided new perspectives on tumor immunization and individualized therapy in GC. Evaluation of the IRTF modulation patterns in individual patients will help to enhance our understanding of immune specificities, and thus, guide rational and personalized therapeutic strategies.

## Data Availability Statement

The datasets presented in this study can be found in online repositories. The names of the repository/repositories and accession number(s) can be found in the article/**Supplementary Material**.

## Ethics Statement

Ethical review and approval was not required for the study on human participants in accordance with the local legislation and institutional requirements. Written informed consent for participation was not required for this study in accordance with the national legislation and the institutional requirements.

## Author Contributions

L-TH and G-JW designed the project. X-YG and X-JG collected the clinical samples and analyzed the data. G-JW wrote the original draft of the manuscript. L-TH, X-FX, and J-FJ reviewed and edited the manuscript. X-FX and J-FJ supervised the research. All authors contributed to the article and approved the submitted version.

## Funding

This work was supported by grants from the third round of public welfare development and reform pilot projects of Beijing Municipal Medical Research Institutes (Beijing Medical Research Institute, 2019-1), the joint fund for key projects of National Natural Science Foundation of China (U20A20371), the National High Technology Research and Development Program of China (863 Program, No. 2014AA020603), “Double First Class” disciplinary development Foundation of Peking University (BMU2019LCKXJ011), the National Natural Science Foundation of China (Nos. 81872502, 81802471, 81972758, 82073312), Capital’s funds for health improvement and research (2018-2-1023), Beijing municipal administration of hospitals’youth program (No.QML20181102), Beijing Municipal Administration of Hospitals Incubating Program (PX2019040), The Research Fund for Young Scholars of Beijing (2018000021469G265), Clinical Medicine Plus X-Young Scholars Project, Peking University (the Fundamental Research Funds for the Central Universities, PKU2020LCXQ001), the Science Foundation of Peking University Cancer Hospital (2020-6, 2020-22).

## Conflict of Interest

The authors declare that the research was conducted in the absence of any commercial or financial relationships that could be construed as a potential conflict of interest.

## Publisher’s Note

All claims expressed in this article are solely those of the authors and do not necessarily represent those of their affiliated organizations, or those of the publisher, the editors and the reviewers. Any product that may be evaluated in this article, or claim that may be made by its manufacturer, is not guaranteed or endorsed by the publisher.
