# Supplementary material for: A Novel Classification and Scoring Method Based on Immune-Related Transcription Factor Regulation Patterns in Gastric Cancer
Source: Front Oncol. 2022 May 17;12:887244. doi: 10.3389/fonc.2022.887244 (PMC9152319; doi:10.3389/fonc.2022.887244)
Supplement: Supplementary file 1 [file DataSheet_1.docx]

Supplementary Figures


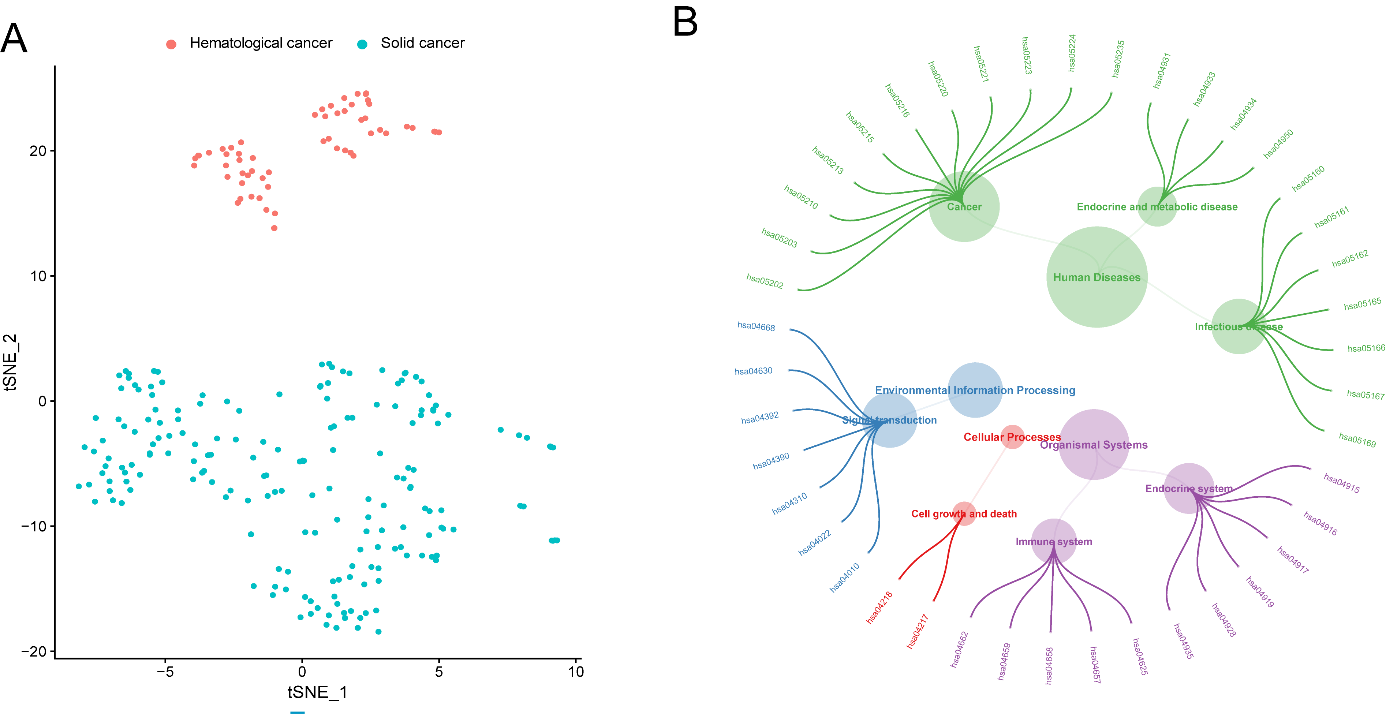


Figure S1: (A) The t-SNE results for the classification of hematological cancer cells and solid cancer cell lines from FANTOM5. (B) KEGG enrichment analysis of IRTFs.


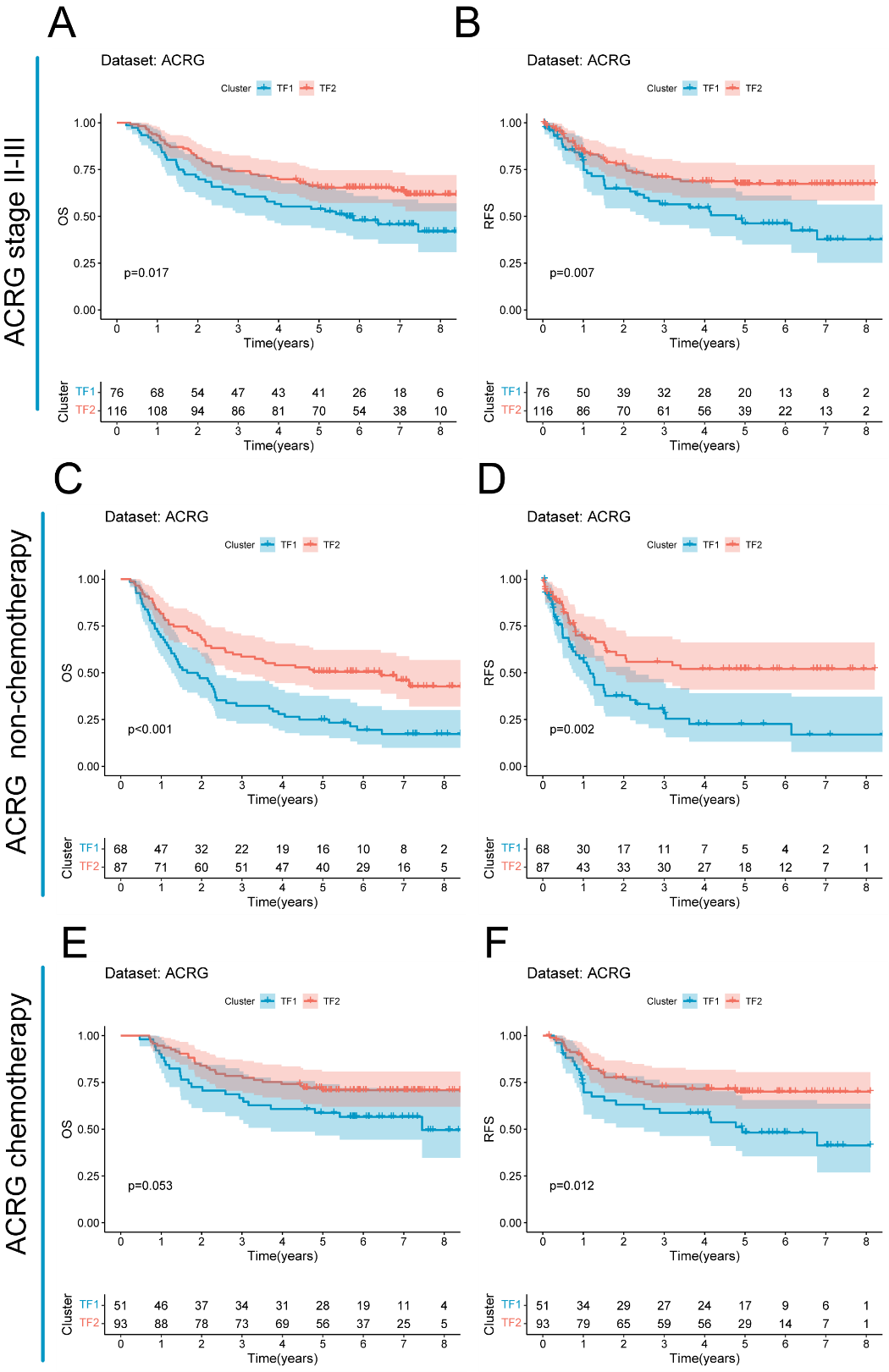


Figure S2: OS and RFS analyses of important clinical phenotypes for the ACRG cohort. (A-B) OS and RFS analyses in stage II-stage III. (C-D) OS and RFS analyses of the non-chemotherapy group. (E-F) OS and RFS analyses of the chemotherapy group.


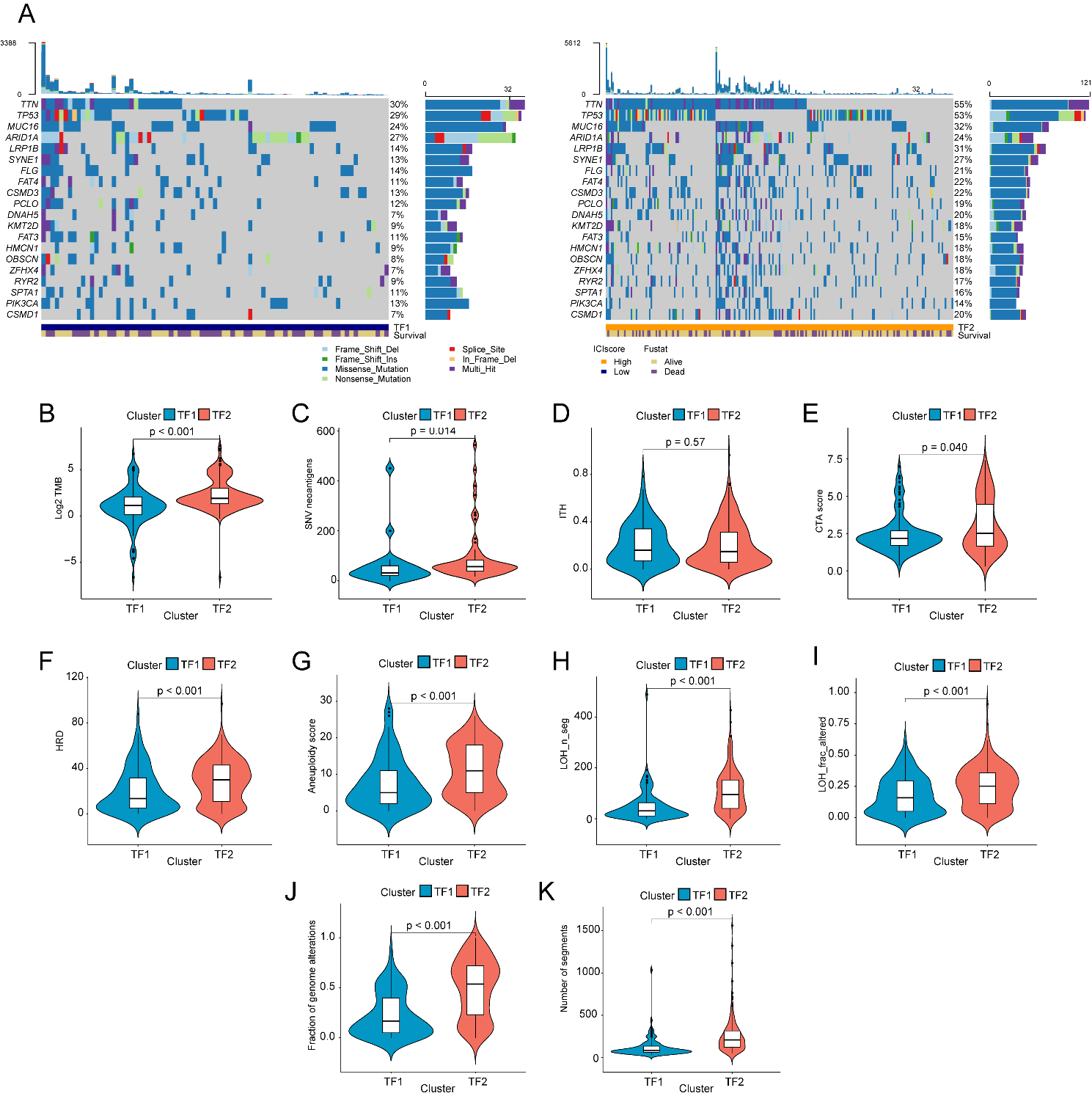


Figure S3: Immunogenicity characteristics for distinct IRTF regulation patterns. (A) The oncoPrint was constructed using TF1 (on the left) and TF2 (on the right). Individual patients are represented in each column. (B-K) Differences in immunogenicity-related indicators for distinct IRTF regulation patterns, including TMB, SNV neoantigens, ITH, CTA score, HRD, aneuploidy score, CNV burden, and loss of heterozygosity.


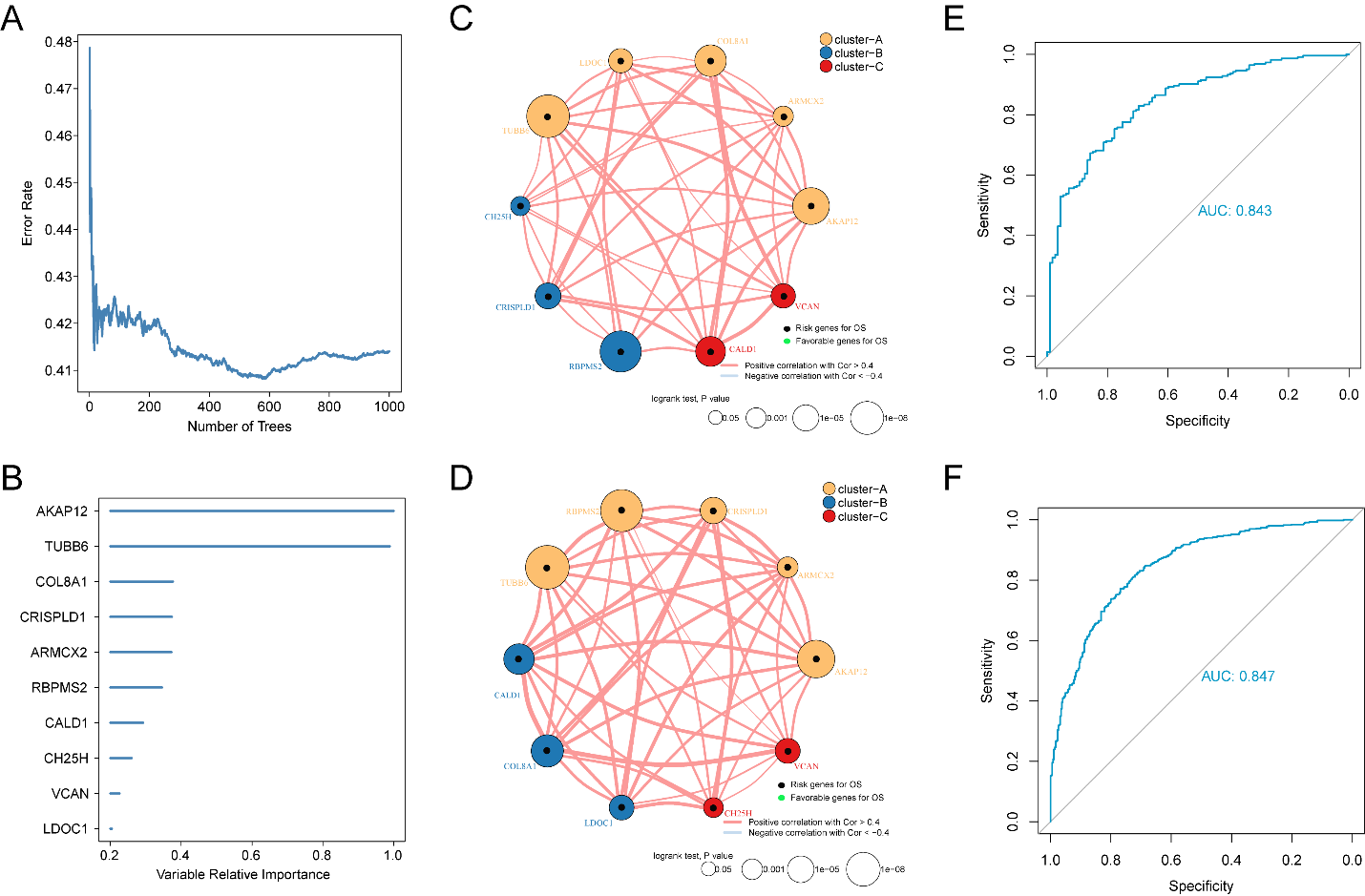


Figure S4: Construction of the IRTF score system. (A) The random forest showed error rates for the data as a function of the classification tree. (B) The relative importance of the characteristic genes. (C-D) The interaction between m6A regulators in the combined cohort (C) and the TCGA-STAD cohort (D). (E-F) The ROC analysis showed that the IRTF score system retained the characteristics of the IRTF regulation patterns in the TCGA-STAD cohort (E) and the combined cohort (F).


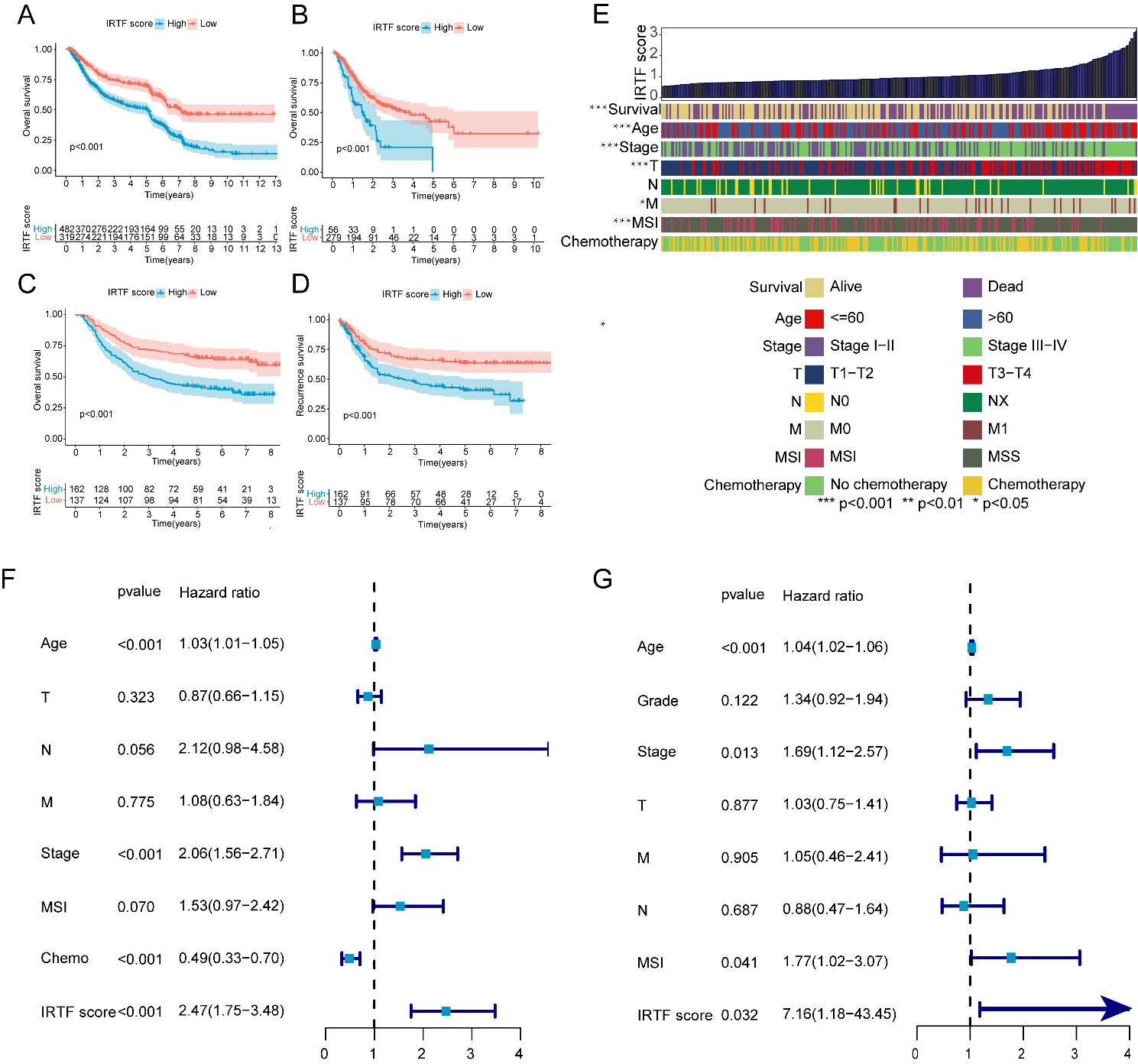


Figure S5: The prognostic value of the IRTF score and correlation between the clinical phenotypes and IRTF scores. (A-B) OS analyses for the high and low IRTF score groups of the combined cohort (A) and the TCGA-STAD cohort (B). (C-D) OS and RFS analyses for the high and low IRTF score groups of the ACRG cohort. (E) Heatmap showing the correlation between the IRTF scores and clinical phenotypes. (F-G) Multivariate Cox regression analysis of the IRTF scores for the ACRG cohort (F) and the TCGA-STAD cohort (G) shown by the forest plot.


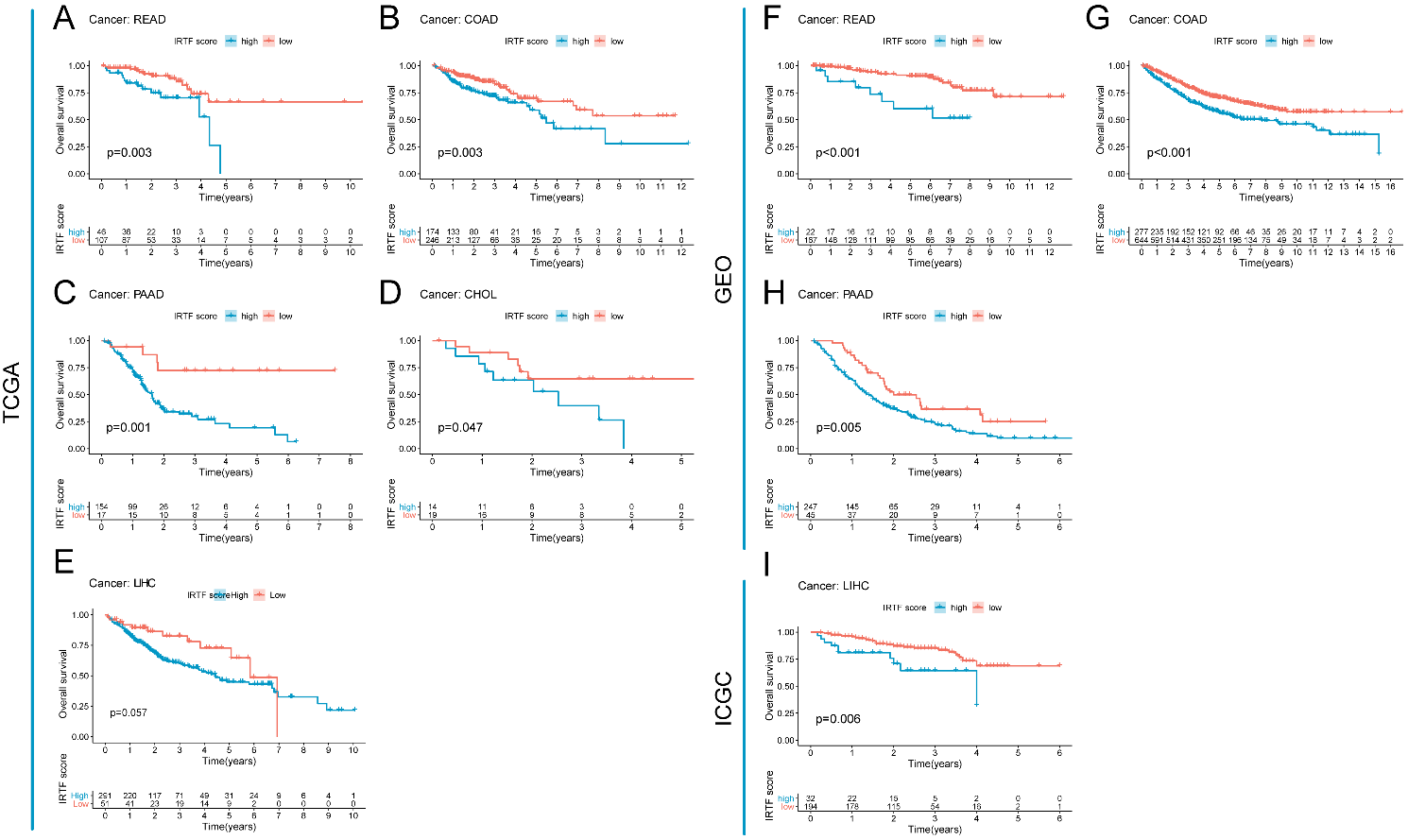


Figure S6: Prognostic value of the IRTF score in multi-cancers. (A-E) OS analyses for the high and low IRTF score groups of the TCGA cohorts, including TCGA-READ, TCGA-COAD, TCGA-PAAD, TCGA-CHOL, and TCGA-LIHC. (F) OS analysis of the IRTF score for the GSE87211 READ cohort. (G) OS analysis of the IRTF score for the GSE38832, GSE17538, and GSE39582 combined COAD cohort. (H) OS analysis of the IRTF score for the GSE28735, GSE62452, GSE71729, and GSE57495 combined PAAD cohort. (I) OS analysis of the IRTF score for the ICGC-LIRI LIHC cohort.
