# Supplementary material for: A Novel Classification and Scoring Method Based on Immune-Related Transcription Factor Regulation Patterns in Gastric Cancer
Source: Front Oncol. 2022 May 17;12:887244. doi: 10.3389/fonc.2022.887244 (PMC9152319; doi:10.3389/fonc.2022.887244)
Supplement: Supplementary file 2 [file DataSheet_2.docx]

Supplementary Methods

**Dataset sources**

**Sources of cell expression data**

First, we obtained RNA-seq expression data (TPM) for immune cells and cancer cell lines of 50 human tissues from FANTOM5 (<https://fantom.gsc.riken.jp/5/>) (1). Hematological cancer cell lines were excluded, as they differ in the expression patterns from solid cancer cell lines (Figure S2A), resulting in the inclusion of 110 immune and 194 cancer cell lines in our study (Table 1). Additionally, we downloaded two single-cell RNA-seq datasets containing tumor and immune cells from the Gene-Expression Omnibus (GEO, https://www.ncbi.nlm.nih.gov/geo/) (GSE75688 (2) and GSE72056 (3)).

**Sources of GC datasets**

Public transcriptome expression data of GC and the corresponding clinical data were obtained from the GEO and The Cancer Genome Atlas (TCGA) (<https://portal.gdc.cancer.gov/>) databases. To ensure the greatest possible overlap of genes between the different datasets, we retained only four datasets based on the GPL570 platform, comprising GSE15459 (4), GSE34942 (5), GSE57303 (6), and GSE62254 (ACRG) (7). Additionally, a cohort (PUCH), with mismatch repair deficiency (dMMR) information from our center, Peking University Cancer Hospital (PUCH), was also included in our study, which was performed using the Agilent human mRNA & lncRNA Array 4.0 platform (8). Patients with no survival information or overall survival ≤ 1 month were removed from further evaluation. Besides, a cohort (ERP107734) from the Sequence Read Archive (SRA) (https://www.ncbi.nlm.nih.gov/sra/), receiving immunotherapy (9), was also included in our study. For all RNAseq datasets, the expression values were transformed to log2 (TPM+1) values. The basic information of all the GC datasets used in this study has been summarized in Table 2. Additionally, the somatic mutation data and copy number variation data were acquired from the TCGA database.

**Source of other cancer datasets**

We collected transcriptome expression data of digestive system cancers from GEO and TCGA, including rectum adenocarcinoma (READ) (TCGA-READ, GSE87211 (10)), colon adenocarcinoma (COAD) (TCGA-COAD, GSE38832 (11), GSE17538 (12), GSE39582 (13)), pancreatic adenocarcinoma (PAAD) (TCGA-PAAD, GSE28735 (14), GSE62452 (15), GSE71729 (16), GSE57495 (17)), cholangiocarcinoma (CHOL) (TCGA-CHOL), liver hepatocellular carcinoma (LIHC) (TCGA-LIHC, ICGC-LIRI). Patients with no survival information or overall survival ≤ 1 month were removed from further evaluation. The basic information of all other cancer datasets from GEO used in this study has been summarized in Table 2.

**Clinical datasets with immune-checkpoint blockade**

In addition to the dataset of GCs treated with anti-PD1 antibody, six cohorts receiving different types of immunotherapies were included in our study. The six cohorts were (1) IMvigor210 (advanced urothelial cancer with anti-PD-L1 antibody) (18), (2) GSE91061 (melanoma with anti-PD1 antibody) (19), (3) GSE78220 (metastatic melanoma with anti-PD1 antibody) (20), (4) PRJEB23709 (melanoma with anti-PD1 antibody or anti-PD1/anti-CTLA4 combined therapy) (21), (5) TCGA: SKCM (advanced melanoma treated with various types of immunotherapies), (6) GSE148476 (chronic lymphocytic leukemia patients with various types of immunotherapies), (7) GSE173839 (breast cancer and patients with neoadjuvant anti-PDL1 antibody)(22), (8) GSE63557 (mouse model treated with anti-CTLA4 antibody) (23), and Pender cohort (pan-cancer treated with various types of immunotherapies)(24) . The basic information of all the immunotherapy datasets has been summarized in Table 2.

**Reference**

1. Lizio M, Harshbarger J, Shimoji H, Severin J, Kasukawa T, Sahin S, Abugessaisa I, Fukuda S, Hori F, Ishikawa-Kato S, Mungall CJ, Arner E, Baillie JK, Bertin N, Bono H, de Hoon M, Diehl AD, Dimont E, Freeman TC, Fujieda K, Hide W, Kaliyaperumal R, Katayama T, Lassmann T, Meehan TF, Nishikata K, Ono H, Rehli M, Sandelin A, Schultes EA, t Hoen PA, Tatum Z, Thompson M, Toyoda T, Wright DW, Daub CO, Itoh M, Carninci P, Hayashizaki Y, Forrest AR, Kawaji H. Gateways to the FANTOM5 promoter level mammalian expression atlas. Genome biology. 2015 Jan 5;16(1):22. eng. Epub 2015/02/28. doi:10.1186/s13059-014-0560-6. Cited in: Pubmed; PMID 25723102.

2. Chung W, Eum HH, Lee HO, Lee KM, Lee HB, Kim KT, Ryu HS, Kim S, Lee JE, Park YH, Kan Z, Han W, Park WY. Single-cell RNA-seq enables comprehensive tumour and immune cell profiling in primary breast cancer. 2017 May 5;8:15081. doi:10.1038/ncomms15081. Cited in: Pubmed; PMID 28474673.

3. Tirosh I, Izar B, Prakadan SM, Wadsworth MH, 2nd, Treacy D, Trombetta JJ, Rotem A, Rodman C, Lian C, Murphy G, Fallahi-Sichani M, Dutton-Regester K, Lin JR, Cohen O, Shah P, Lu D, Genshaft AS, Hughes TK, Ziegler CG, Kazer SW, Gaillard A, Kolb KE, Villani AC, Johannessen CM, Andreev AY, Van Allen EM, Bertagnolli M, Sorger PK, Sullivan RJ, Flaherty KT, Frederick DT, Jané-Valbuena J, Yoon CH, Rozenblatt-Rosen O, Shalek AK, Regev A, Garraway LA. Dissecting the multicellular ecosystem of metastatic melanoma by single-cell RNA-seq. Science (New York, NY). 2016 Apr 8;352(6282):189-96. eng. Epub 2016/04/29. doi:10.1126/science.aad0501. Cited in: Pubmed; PMID 27124452.

4. Ooi CH, Ivanova T, Wu J, Lee M, Tan IB, Tao J, Ward L, Koo JH, Gopalakrishnan V, Zhu Y, Cheng LL, Lee J, Rha SY, Chung HC, Ganesan K, So J, Soo KC, Lim D, Chan WH, Wong WK, Bowtell D, Yeoh KG, Grabsch H, Boussioutas A, Tan P. Oncogenic pathway combinations predict clinical prognosis in gastric cancer. PLoS genetics. 2009 Oct;5(10):e1000676. eng. Epub 2009/10/03. doi:10.1371/journal.pgen.1000676. Cited in: Pubmed; PMID 19798449.

5. Chia NY, Deng N, Das K, Huang D, Hu L, Zhu Y, Lim KH, Lee MH, Wu J, Sam XX, Tan GS, Wan WK, Yu W, Gan A, Tan AL, Tay ST, Soo KC, Wong WK, Dominguez LT, Ng HH, Rozen S, Goh LK, Teh BT, Tan P. Regulatory crosstalk between lineage-survival oncogenes KLF5, GATA4 and GATA6 cooperatively promotes gastric cancer development. Gut. 2015 May;64(5):707-19. eng. Epub 2014/07/24. doi:10.1136/gutjnl-2013-306596. Cited in: Pubmed; PMID 25053715.

6. Qian Z, Zhu G, Tang L, Wang M, Zhang L, Fu J, Huang C, Fan S, Sun Y, Lv J, Dong H, Gao B, Su X, Yu D, Zang J, Zhang X, Ji J, Ji Q. Whole genome gene copy number profiling of gastric cancer identifies PAK1 and KRAS gene amplification as therapy targets. Genes, chromosomes & cancer. 2014 Nov;53(11):883-94. eng. Epub 2014/06/18. doi:10.1002/gcc.22196. Cited in: Pubmed; PMID 24935174.

7. Cristescu R, Lee J, Nebozhyn M, Kim KM, Ting JC, Wong SS, Liu J, Yue YG. Molecular analysis of gastric cancer identifies subtypes associated with distinct clinical outcomes. 2015 May;21(5):449-56. doi:10.1038/nm.3850. Cited in: Pubmed; PMID 25894828.

8. Huang HC, Wen XZ, Xue H, Chen RS, Ji JF, Xu L. Phosphoglucose isomerase gene expression as a prognostic biomarker of gastric cancer. Chinese journal of cancer research = Chung-kuo yen cheng yen chiu. 2019 Oct;31(5):771-784. eng. Epub 2019/12/10. doi:10.21147/j.issn.1000-9604.2019.05.07. Cited in: Pubmed; PMID 31814681.

9. Kim ST, Cristescu R. Comprehensive molecular characterization of clinical responses to PD-1 inhibition in metastatic gastric cancer. 2018 Sep;24(9):1449-1458. doi:10.1038/s41591-018-0101-z. Cited in: Pubmed; PMID 30013197.

10. Hu Y, Gaedcke J, Emons G, Beissbarth T, Grade M, Jo P, Yeager M, Chanock SJ, Wolff H, Camps J, Ghadimi BM, Ried T. Colorectal cancer susceptibility loci as predictive markers of rectal cancer prognosis after surgery. 2018 Mar;57(3):140-149. doi:10.1002/gcc.22512. Cited in: Pubmed; PMID 29119627.

11. Tripathi MK, Deane NG, Zhu J, An H, Mima S, Wang X, Padmanabhan S, Shi Z, Prodduturi N, Ciombor KK, Chen X, Washington MK, Zhang B, Beauchamp RD. Nuclear factor of activated T-cell activity is associated with metastatic capacity in colon cancer. Cancer research. 2014 Dec 1;74(23):6947-57. eng. Epub 2014/10/17. doi:10.1158/0008-5472.can-14-1592. Cited in: Pubmed; PMID 25320007.

12. Smith JJ, Deane NG, Wu F, Merchant NB, Zhang B, Jiang A, Lu P, Johnson JC, Schmidt C, Bailey CE, Eschrich S, Kis C, Levy S, Washington MK, Heslin MJ, Coffey RJ, Yeatman TJ, Shyr Y, Beauchamp RD. Experimentally derived metastasis gene expression profile predicts recurrence and death in patients with colon cancer. Gastroenterology. 2010 Mar;138(3):958-68. eng. Epub 2009/11/17. doi:10.1053/j.gastro.2009.11.005. Cited in: Pubmed; PMID 19914252.

13. Marisa L, de Reyniès A, Duval A, Selves J, Gaub MP, Vescovo L, Etienne-Grimaldi MC, Schiappa R, Guenot D, Ayadi M, Kirzin S, Chazal M, Fléjou JF, Benchimol D, Berger A, Lagarde A, Pencreach E, Piard F, Elias D, Parc Y, Olschwang S, Milano G, Laurent-Puig P, Boige V. Gene expression classification of colon cancer into molecular subtypes: characterization, validation, and prognostic value. PLoS medicine. 2013;10(5):e1001453. eng. Epub 2013/05/24. doi:10.1371/journal.pmed.1001453. Cited in: Pubmed; PMID 23700391.

14. Zhang G, Schetter A, He P, Funamizu N, Gaedcke J, Ghadimi BM, Ried T, Hassan R, Yfantis HG, Lee DH, Lacy C, Maitra A, Hanna N, Alexander HR, Hussain SP. DPEP1 inhibits tumor cell invasiveness, enhances chemosensitivity and predicts clinical outcome in pancreatic ductal adenocarcinoma. PloS one. 2012;7(2):e31507. eng. Epub 2012/03/01. doi:10.1371/journal.pone.0031507. Cited in: Pubmed; PMID 22363658.

15. Yang S, He P, Wang J, Schetter A, Tang W, Funamizu N, Yanaga K, Uwagawa T, Satoskar AR, Gaedcke J, Bernhardt M, Ghadimi BM, Gaida MM, Bergmann F, Werner J, Ried T, Hanna N, Alexander HR, Hussain SP. A Novel MIF Signaling Pathway Drives the Malignant Character of Pancreatic Cancer by Targeting NR3C2. Cancer research. 2016 Jul 1;76(13):3838-50. eng. Epub 2016/05/20. doi:10.1158/0008-5472.can-15-2841. Cited in: Pubmed; PMID 27197190.

16. Moffitt RA, Marayati R, Flate EL, Volmar KE, Loeza SG, Hoadley KA, Rashid NU, Williams LA, Eaton SC, Chung AH, Smyla JK, Anderson JM, Kim HJ, Bentrem DJ, Talamonti MS, Iacobuzio-Donahue CA. Virtual microdissection identifies distinct tumor- and stroma-specific subtypes of pancreatic ductal adenocarcinoma. 2015 Oct;47(10):1168-78. doi:10.1038/ng.3398. Cited in: Pubmed; PMID 26343385.

17. Chen DT, Davis-Yadley AH, Huang PY, Husain K, Centeno BA, Permuth-Wey J, Pimiento JM, Malafa M. Prognostic Fifteen-Gene Signature for Early Stage Pancreatic Ductal Adenocarcinoma. PloS one. 2015;10(8):e0133562. eng. Epub 2015/08/08. doi:10.1371/journal.pone.0133562. Cited in: Pubmed; PMID 26247463.

18. Mariathasan S, Turley SJ, Nickles D, Castiglioni A, Yuen K, Wang Y, Kadel EE, III, Koeppen H, Astarita JL, Cubas R, Jhunjhunwala S, Banchereau R, Yang Y, Guan Y, Chalouni C, Ziai J, Şenbabaoğlu Y, Santoro S, Sheinson D, Hung J, Giltnane JM, Pierce AA, Mesh K, Lianoglou S, Riegler J, Carano RAD, Eriksson P, Höglund M, Somarriba L, Halligan DL, van der Heijden MS, Loriot Y, Rosenberg JE, Fong L, Mellman I, Chen DS, Green M, Derleth C, Fine GD, Hegde PS, Bourgon R, Powles T. TGFβ attenuates tumour response to PD-L1 blockade by contributing to exclusion of T cells. Nature. 2018 Feb 22;554(7693):544-548. eng. Epub 2018/02/15. doi:10.1038/nature25501. Cited in: Pubmed; PMID 29443960.

19. Riaz N, Havel JJ, Makarov V, Desrichard A, Urba WJ, Sims JS, Hodi FS, Martín-Algarra S, Mandal R, Sharfman WH, Bhatia S, Hwu WJ, Gajewski TF, Slingluff CL, Jr., Chowell D, Kendall SM, Chang H, Shah R, Kuo F, Morris LGT, Sidhom JW, Schneck JP, Horak CE, Weinhold N, Chan TA. Tumor and Microenvironment Evolution during Immunotherapy with Nivolumab. Cell. 2017 Nov 2;171(4):934-949.e16. eng. Epub 2017/10/17. doi:10.1016/j.cell.2017.09.028. Cited in: Pubmed; PMID 29033130.

20. Hugo W, Zaretsky JM, Sun L, Song C, Moreno BH, Hu-Lieskovan S, Berent-Maoz B, Pang J, Chmielowski B, Cherry G, Seja E, Lomeli S, Kong X, Kelley MC, Sosman JA, Johnson DB, Ribas A, Lo RS. Genomic and Transcriptomic Features of Response to Anti-PD-1 Therapy in Metastatic Melanoma. Cell. 2016 Mar 24;165(1):35-44. eng. Epub 2016/03/22. doi:10.1016/j.cell.2016.02.065. Cited in: Pubmed; PMID 26997480.

21. Gide TN, Quek C, Menzies AM, Tasker AT, Shang P, Holst J, Madore J, Lim SY, Velickovic R, Wongchenko M, Yan Y, Lo S, Carlino MS, Guminski A, Saw RPM, Pang A, McGuire HM, Palendira U, Thompson JF, Rizos H, Silva IPD, Batten M, Scolyer RA, Long GV, Wilmott JS. Distinct Immune Cell Populations Define Response to Anti-PD-1 Monotherapy and Anti-PD-1/Anti-CTLA-4 Combined Therapy. Cancer cell. 2019 Feb 11;35(2):238-255.e6. eng. Epub 2019/02/13. doi:10.1016/j.ccell.2019.01.003. Cited in: Pubmed; PMID 30753825.

22. Pusztai L, Yau C, Wolf DM, Han HS, Du L, Wallace AM, String-Reasor E, Boughey JC, Chien AJ, Elias AD, Beckwith H, Nanda R, Albain KS, Clark AS, Kemmer K, Kalinsky K, Isaacs C, Thomas A, Shatsky R, Helsten TL, Forero-Torres A, Liu MC, Brown-Swigart L, Petricoin EF, Wulfkuhle JD, Asare SM, Wilson A, Singhrao R, Sit L, Hirst GL, Berry S, Sanil A, Asare AL, Matthews JB, Perlmutter J, Melisko M, Rugo HS, Schwab RB, Symmans WF, Yee D, Van't Veer LJ, Hylton NM, DeMichele AM, Berry DA, Esserman LJ. Durvalumab with olaparib and paclitaxel for high-risk HER2-negative stage II/III breast cancer: Results from the adaptively randomized I-SPY2 trial. Cancer cell. 2021 Jul 12;39(7):989-998.e5. eng. Epub 2021/06/19. doi:10.1016/j.ccell.2021.05.009. Cited in: Pubmed; PMID 34143979.

23. Lesterhuis WJ, Rinaldi C, Jones A, Rozali EN, Dick IM, Khong A, Boon L, Robinson BW, Nowak AK, Bosco A, Lake RA. Network analysis of immunotherapy-induced regressing tumours identifies novel synergistic drug combinations. Scientific reports. 2015 Jul 21;5:12298. eng. Epub 2015/07/22. doi:10.1038/srep12298. Cited in: Pubmed; PMID 26193793.

24. Pender A, Titmuss E, Pleasance ED, Fan KY, Pearson H, Brown SD, Grisdale CJ, Topham JT, Shen Y, Bonakdar M, Taylor GA, Williamson LM, Mungall KL, Chuah E, Mungall AJ, Moore RA, Lavoie JM, Yip S, Lim H, Renouf DJ, Sun S, Holt RA, Jones SJM, Marra MA, Laskin J. Genome and Transcriptome Biomarkers of Response to Immune Checkpoint Inhibitors in Advanced Solid Tumors. Clinical cancer research : an official journal of the American Association for Cancer Research. 2021 Jan 1;27(1):202-212. eng. Epub 2020/10/07. doi:10.1158/1078-0432.Ccr-20-1163. Cited in: Pubmed; PMID 33020056.
